# The importance of the Andes in the evolutionary radiation of Sigmodontinae (Rodentia, Cricetidae), the most diverse group of mammals in the Neotropics

**DOI:** 10.1038/s41598-023-28497-0

**Published:** 2023-02-07

**Authors:** Paulo Vallejos-Garrido, Kateryn Pino, Nicolás Espinoza-Aravena, Alexander Pari, Oscar Inostroza-Michael, Macarena Toledo-Muñoz, Boris Castillo-Ravanal, Viviana Romero-Alarcón, Cristián E. Hernández, R. Eduardo Palma, Enrique Rodríguez-Serrano

**Affiliations:** 1 Programa de Doctorado en Sistemática y Biodiversidad, Facultad de Ciencias Naturales y Oceanográficas, Universidad de Concepción, Concepción, Chile; 2 Programa de Magíster en Ciencias Mención Zoología, Facultad de Ciencias Naturales y Oceanográficas, Universidad de Concepción, Concepción, Chile; 3 Laboratorio de Mastozoología, Departamento de Zoología, Facultad de Ciencias Naturales y Oceanográficas, Universidad de Concepción, Concepción, Chile; 4 Laboratorio de Ecología Evolutiva y Filoinformática, Departamento de Zoología, Facultad de Ciencias Naturales y Oceanográficas, Universidad de Concepción, Concepción, Chile; 5 Museo de Historia Natural, Universidad Nacional de San Agustín de Arequipa, Arequipa, Perú; 6 Vida Silvestre Investigadores Limitada, Concepción, Chile; 7 Universidad Católica de Santa María, Arequipa, Perú; 8 Laboratorio de Biología Evolutiva, Departamento de Ecología, Facultad de Ciencias Biológicas, Pontificia Universidad Católica de Chile, Santiago, Chile; 9 Universidad Católica de la Santísima Concepción, Concepción, Chile

**Keywords:** Biogeography, Speciation

## Abstract

The Andean mountains stand out for their striking species richness and endemicity that characterize many emblematic Neotropical clades distributed in or around these mountains. The radiation of the Sigmodontinae subfamily, the most diversified mammalian group in the Neotropics, has been historically related to Andean orogenesis. We aim to evaluate this interplay between geological processes and biological responses through the diversification dynamics, the biogeographical history, and the range evolution of the subfamily. For these, we built the most comprehensive phylogeny and gathered 14,836 occurrences for the subfamily. We identified one shift in the speciation rate in the genus *Akodon*, which suffered their Andean radiation after the arrival of non-Andean ancestors. Our biogeographic analyses show multiple dispersal paths throughout the evolution that allowed this subfamily to colonize all Neotropics. The Northern Andes and Central-Southern Andes were the most important sources of diversity. In addition, the Central-Southern Andes were the most relevant sink, receiving the highest number of lineages. The Andean region exhibited higher speciation and turnover rates than non-Andean regions. Thus, our results support the crucial role of the Andean Mountains in the Sigmodontinae radiation, acting as a "macroevolutionary cradle" and "species attractor" for several sigmodontine lineages at different times, and as a "species pump" becoming the biogeographic source of multiple widely distributed neotropical lineages. Then, complex macroevolutionary dynamics would explain these rodents' high extant Andean diversity and their wide distribution in the Neotropics.

## Introduction

The extant biodiversity shows a strikingly spatial pattern of extraordinary concentration in high-altitude geographic areas, i.e., in regions on Earth where tectonic movements have developed large mountain ranges^1–7^. Mountains host a quarter of all species on Earth in less than a tenth of their surface area, making them the most diverse regions on the planet^7,8^. For example, tropical mountains are hotspots of biodiversity and endemism^9^. The mountain formation drastically transforms a previously homogeneous and geologically static landscape into a climatically and topographically heterogeneous region, creating habitats where lineages from surrounding lands arrive and diversify^8,10,11^. New comparative approaches show that the underlying effect of mountain origin on diversification is the interplay between long-distance dispersal and local recruitment, followed by adaptation and speciation through interaction with the landscape, climate, and environment changes^9,12,13^.

Accordingly, Chazot et al.^14^ proposed two biogeographical scenarios for the origin of high diversity in mountain systems. The first hypothesis suggests that mountains act as a "macroevolutionary cradle": a geographical area that displays a particular set of characteristics that maximize the potential for diversification^14–16^. Thus, mountain lineages can attain the highest speciation rates responding to different environments in an altitudinal gradient by parapatric speciation^17–22^. Furthermore, higher diversification events could be due to vicariant allopatric differentiation between mountain-valleys^6,23,24^, and vicariant peripatric speciation^25^. The second hypothesis proposes that mountains are "species attractors"^14,26^. They would function as bridges or secondary contact zones, allowing the arrival of non-mountain lineages by dispersal events from lowlands through its environmentally diverse slopes^27–30^. Additionally, mountain uplifts generate changes in adjacent regional biomes and biota due to gradual and abrupt environmental changes accompanying its building^5,8,30^. Then, a mountain may also act as a "species pump"^14,16,32–34^. That is, they originate lineages that potentially colonize the surrounding lowland areas^9,35–38^.

The direct and indirect consequences of mountain uplifts on biodiversity diversification are particularly complex to decipher in areas where these effects cover a wide range of climate zones, such as the Andes in South America^39,40^. The Andean mountains are striking for their high species richness and endemicity^1,41,42^ and the rapid diversification rates that characterize many of their emblematic neotropical clades^31,38,40,43–48^. The sedimentary records from the Andean foreland basins indicate that uplift in the Andes initiated in the Late Cretaceous: ~ 100 million years ago (Ma) in Patagonia and ~ 70 Ma in the central and northern Andes^49–51^. However, most of the high species richness in these areas, seems to be the result of recent and rapid species diversification, coincident with the reaccelerated uplift of some Andean domains over the past 15 million years^39–41,51–54^. Thus, most of the Andean diversity would have originated after or in synchrony with the rise of these significant and recent Andean uplift pulses. In this sense, a similar pattern of recent temporal concordance with the Andean uplift seems to be the radiation of the rodent subfamily Sigmodontinae^55^, the most diversified mammal group in the Neotropics, with 489 species and 13 tribe-level lineages^56–59^. The foundational research of Reig^60,61^ proposed the Andes as the center of origin of the Sigmodontinae, being the main region for their diversification. Nevertheless, alternative scenarios of origin and radiation for sigmodontines have been proposed, such as an origin outside the Andes, at least for most tribes^62^, or even outside of South America^57,63–66^. Thus, the origin of sigmodontine rodents still needs to be clarified^67^. Currently, sigmodontine rodents are mainly distributed in South America and show the highest species richness in high elevations of the Central Andes, followed by the Brazilian highlands^58,62,68^. The glittery recent ecological and geographical spread of Sigmodontinae in South America and its high extant species accumulation pattern in the Andean region results from an exceptional ecological opportunity on a continental scale, being the most rapid geographically discrete diversification event in mammals^58^. Therefore, this group is an ideal vertebrate model to study the relationship between the Andean surface uplift, diversification, and the notable species accumulation in the Andean regions^55,57,58,61,64,69^. In this study, we aim to evaluate the effect of the Andean mountains on the diversification dynamics and biogeographical history of the Sigmodontinae. Therefore, we will establish the relative contribution of the Andes to the diversity of this rodent group that can be attributed to a cradle of diversity, species attractor, species pump, or a combination of them.

## Methods

### Biogeographic data collection

We used 12 of 14 biogeographic regions proposed by Schenk and Steppan^58^ focused on sigmodontine rodents. These areas were divided based on geographic features (e.g., Andes, Isthmus of Panama, Galápagos Islands), relatively abrupt changes in habitats, and previous regionalization studies^70–72^. The biogeographic regions are Mesoamerica, Guinean savanna and Antillean Islands, Chocó and Tumbes, Northern Andes, Central and Southern Andes, Amazonia, West South American coastal, Cerrado and Caatinga, Atlantic Forest, Chaco, Patagonian Steppe, and Galápagos (Fig. 2). Here, we handle the Andean Region as two areas, considering the Central Andes, Southern Andes, and the Altiplano as one region. For this, we used the Olson et al.^71^ shapefile with the well-defined regions available at https://www.worldwildlife.org/publications/terrestrial-ecoregions-of-the-world.

To define species presence-absence in each biogeographic region, we digitalized 14,836 occurrences for 387 sigmodontine rodents. Occurrence data were obtained mainly from Patton et al.^73^. For species not included in Patton et al.^73^, primarily species distributed out of South America and for those recent taxonomic arrangements and new species descriptions, we reviewed specialized literature or obtained them from GBIF (https://gbif.org; retrieved in January 2022) (Supplementary Information, Table S1). We used only the GBIF occurrences with preserved specimen vouchers. The duplicate occurrences were deleted from the final dataset. Finally, we contrasted each occurrence with the updated mammal species distribution maps from Map of Life [^74^; https://mol.org/species/] to adjust and correct possible bias occurrences.

### Phylogenetic analyses

#### The Sigmodontinae’s phylogeny

To evaluate the biogeographic evolutionary history of sigmodontine rodents, we first estimated a multigene calibrated phylogeny for 387 of 489 Sigmodontinae species, representing 79% of the known present species diversity [^56^; MDD_v1.9, https://www.mammaldiversity.org/]. To our knowledge, this phylogeny represents the most comprehensive phylogeny of the subfamily. We compiled a matrix alignment of 11 gene fragments from two mitochondrial and nine nuclear genes for a total length of 11,472 base pairs (bp) (Supplementary Information file 1, Table S2). The matrix was compiled from Maestri et al.^58^ and Shenck and Steppan^62^ databases, then we updated and curated it according to the current taxonomy^56^. We contrasted each sequence in those databases and added new ones based on recent phylogenetic published studies at low taxonomic ranges (i.e., species complex, genera, and tribes) (See references cited in the Supplementary information File 4). Thus, our matrix included 108 and 96 more species than Maestri et al.^58^ and Shenck and Steppan^62^, respectively. Sequence alignments were performed using the MUSCLE algorithm^75^ in MEGA version 11^76^. Additionally, we included 19 species from the other subfamilies of the Cricetidae family (Tylominae, Neotominae Arvicolinae, and Cricetinae) and two species from the Muridae family to root the tree^77,78^. As higher-level phylogenetic relationships (i.e., tribes) of Sigmodontinae are poorly resolved using genes sequences^78–80^, we included the genomic data set alignment of 2958 ultraconserved elements (UCEs) of 60 species (53 sigmodontines) from Parada et al.^77^ to our multigene matrix (Supplementary Information file2). Based on the total alignment matrix (UCEs and multigene data), we inferred the phylogenetic hypothesis of sigmodontines relationships under the maximum likelihood criterion using IQTREE 1.6.12^81^. We performed a partitioned analysis^82^, considering each locus as an individual partition (− p), and their corresponding best-fit nucleotide substitution model was selected by the ModelFinder algorithm^83^ (− m MF), under the Bayesian Inference Criterion (BIC). In addition, we perform a full tree search for every model (− mtree) to get an accurate analysis. We obtained the maximum likelihood (ML) tree using a search of 1000 iterations (− 1000), without constraint nodes, and support analysis using 100 replicates of nonparametric bootstrap (− b 100). We fixed the seed in 999 (− seed 999) to generate a reproducible analysis.

#### Chronology for Sigmodontinae

We estimated the divergence times in BEAST v2.6.7^84^ under the Fossilized Birth–Death (FBD) model as implemented in the Sample Ancestor package^85,86^. This calibration method considers fossil and extant species as the result of the same diversification process, avoiding arbitrary and problematic calibration densities commonly utilized in techniques such as node calibration. Also, fossil taxa can be treated as direct ancestors or extinct tips, accommodating their topological placement according to an MCMC algorithm^86^. We selected an optimized relaxed clock with the ORC package^87^. The models of nucleotide evolution for each molecular partition were simultaneously estimated along with the phylogenetic inference according to the Bayesian algorithm implemented in the package bModelTest^88^. The ORC and bModelTest packages are implemented in BEAST 2. We used a multigene alignment matrix of 11,472 bp to estimate divergence times along 25 fossil taxa (23 sigmodontines) with ages ranging from 13.6 to 0.01 Ma to infer the divergence times (Supplementary Information, Table S3). The uncertainty of fossil taxa ages was considered part of the FBD model inference by incorporating the ages distribution as priors^89^. We used taxonomic information following Ronez et al.^67^ to restrict the placement of extinct taxa in the phylogeny to the most exclusive taxonomic level possible (i.e., tribes) (Supplementary Information, Table S3). We ran 157 million MCMC iterations, sampling every 10 000 iterations, and examined the convergence and effective sample sizes (ESS) in Tracer v1.7.2^90^. Next, we removed the fossils from the resulting sample trees using the FullToExtantTreeConverter package implemented in BEAST 2. Finally, we estimated the maximum clade credibility (MCC) tree after excluding the first 10% of sample trees as burn-in (Supplementary information file3). We used the MCC tree for the downstream diversification and biogeographic analyses.

### Diversification analyses

To infer the diversification dynamics of sigmodontine rodents, we estimated branch-specific speciation rates in ClaDS^91^. We used the model with constant turnover ε (i.e., constant ratio between extinction and speciation rates; ClaDS2) and ran a newly developed ClaDS algorithm based on data augmentation techniques which enables us to estimate mean rates through time^92^. Three independent chains were run, and their convergence was checked using a Gelman–Rubin diagnostic criterion^93^. We recorded (1) the estimated hyperparameters (α, σ, ε) and the value m = α × exp(σ^2/2^), which indicates the general trend of the sigmodontine speciation rates through time^91^, and (2) the lineage-specific speciation rates by tribe and by distribution (Andean, non-Andean and widespread). Also, we used a time-dependent model implemented in BAMM v.2.5.0^94^ to estimate speciation rates across the phylogeny and their variation through time and among lineages. First, we account for incomplete taxon sampling by assigning a non-random incomplete taxa sampling method^94^ (we incorporated sampling fractions of each genus sampled according to Burgin et al. [^56^; https://www.mammaldiversity.org/] (Supplementary Information, Table S4). Next, we explicitly set the priors for the analysis of the calibrated Sigmodontinae phylogeny obtained in the phylogenetic analysis using the ‘setBAMMpriors’ function of the ‘BAMMtools’ R-package^94^. Then, we ran four Markov Chains Monte Carlo (MCMC) with 100 million generations, sampling parameters every 10,000 generations. We reviewed the convergence of the runs and parameters with the effective sample size (all the ESS values > 200), and the first 20% of the sampled data was discarded as burn‐in. Next, we examined the 95% credible set of macroevolutionary shift configurations and the best set of rate shifts using posterior probability (Supplementary Information, Figs. S2, S3; Table S5). Finally, diversification rates and rate shift configurations were plotted using the ‘BAMMtools’ R-package^94^.

### Biogeographic analyses

To infer the geographical range evolution of sigmodontine rodents and the frequency, time, and direction of the dispersal events between biogeographic regions, we test the Dispersal–Extinction–Cladogenesis model of range evolution [DEC^95,96^] and DEC + *j* model, which allowed for ‘founder event’ speciation^97,98^, both implemented in the R-package BioGeoBEARS 1.1.2^99^. We choose the best-fitting model based on the likelihood, Akaike Information Criterion (AIC), and AICc weight (Supplementary information, Table S7). We used the single-most probable state (geographic range) at each node estimated with the selected best-fitting biogeographic model (DEC, log-likelihood = − 1279) to estimate dispersal events following the approach proposed by Antonelli et al.^8^. Thus, we computed the absolute number of dispersal events through time by extracting the areas and ages of all nodes from the phylogeny. In addition, we calculated relative numbers of dispersal events by dividing absolute numbers by the total length of all branches within each 2-My time bin to consider the potential effect of the branch lengths in the number of dispersal events^8,13,100^. Although we did not constrain any a priori dispersal multiplier nor stratify dispersal rates across the phylogeny, the unconstrained model carries fewer assumptions, thus decreasing the risk of manually influencing the results^101^. Dispersal events analyses were performed on the sigmodontine phylogeny only (outgroup removed) and based on Antonelli’s R script ^8^ (Supplementary Information Tables S9, S10).

### Andean effect on diversification rates

To evaluate the impact of Andean or non-Andean regions on Sigmodontinae diversification, we estimate state-specific diversification rates (i.e., each biogeographic region) on sigmodontine phylogeny. We used the GeoHiSSE model^102^ implemented in the GeoHiSSE function of the HISSE v.1.9.6 R-package^103^. This model includes "concealed traits" and is less prone to false positives^102^. GeoHiSSE estimates speciation and extinction rates dependent on geographical trait states and transition rates among states while allowing for widespread distributed ancestors and considering sampling frequencies. Thus, we investigated whether diversification dynamics are associated with geographic ranges by fitting a set of 13 models from the GeoSSE and GeoHiSSE modeling framework to the phylogenetic tree and range data of sigmodontine rodents. We used a uniform taxon sampling for the 33% of species distributed in the Andean region, 47% for the non-Andean region, and 20% in widespread areas (i.e., the species’ distribution includes at least a part of Andean and non-Andean regions; Supplementary information, Table S8). Models vary according to whether geographic range and diversification rate are linked, how they treat extirpation as a process separate from range contraction, and whether the model employs hidden states to allow for additional diversification rate variation that is not linked to geographic range (Table 2). Then, we averaged estimates of diversification parameters and ancestral ranges across 13 models using AIC weights. This approach integrates estimation from each model in proportion to how much the model can explain the pattern in the observed data. Model averaging largely alleviates the subjectivity of choosing thresholds to rank models and permits estimating parameters considering the uncertainty in model fit^102^. We used a model-averaging procedure to summarize states and rates for every model in the set across a given tree.

## Results

### Diversification rates pattern

**Figure 1:**
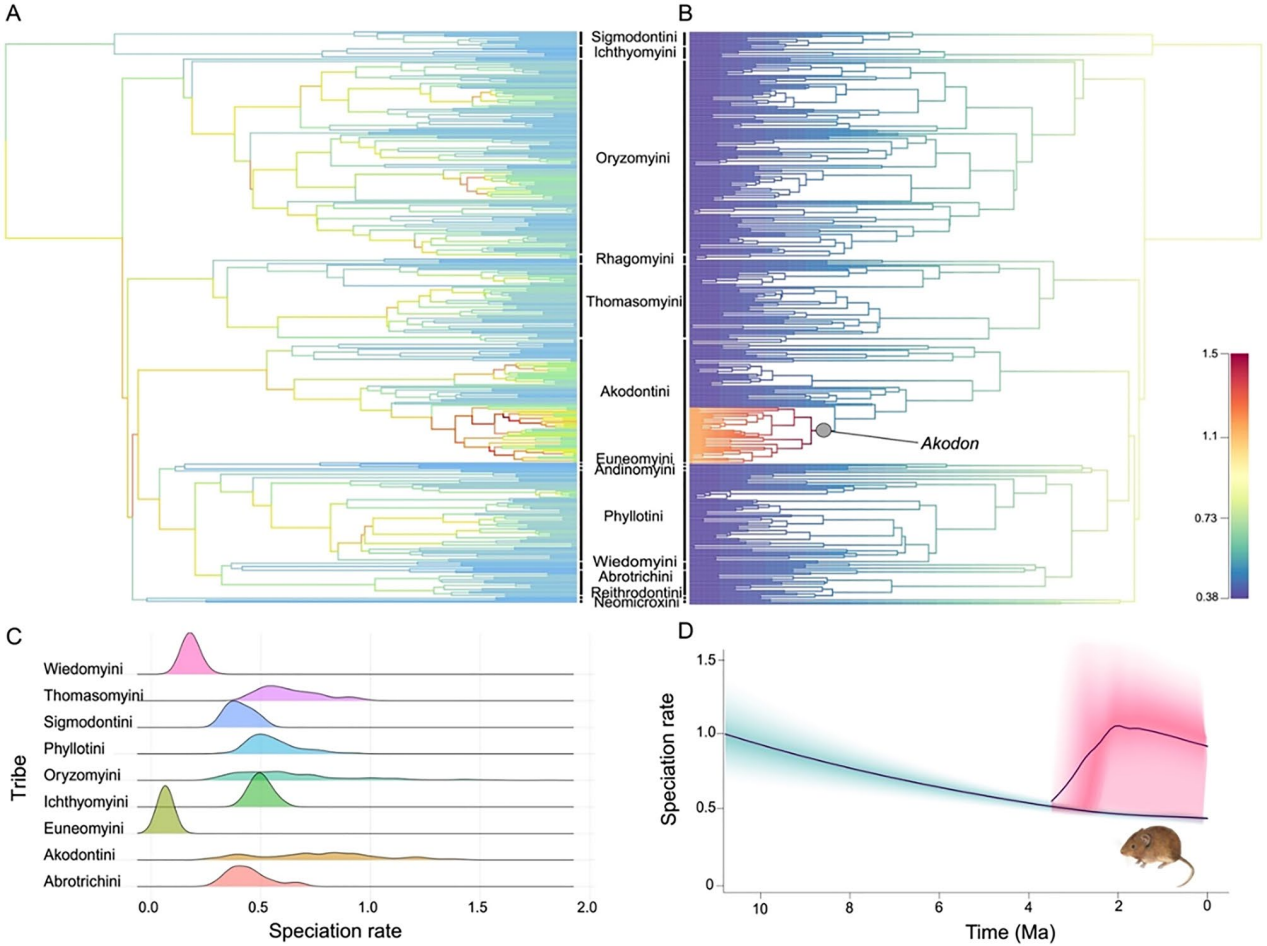
Macroevolutionary dynamics during the radiation of sigmodontine rodents. (**A**) Branch-specific speciation rates under the ClaDS2 model. (**B**) Speciation rate plot (mean phylorate) under BAMM model. The gray circle along the branch leading to the clade of the *Akodon* genus shows the best configuration shift identified by BAMM. Color intensity across branches is proportional to changes in speciation rate. (**C**) Branch-specific speciation rates under the ClaDS2 model by tribe. (**D**) Speciation-through-time trajectories for the Sigmodontinae subfamily (in green) and *Akodon* genus (in red). Illustration of *Akodon* rodent by Alayda Arce-Merma.

**Figure 2:**
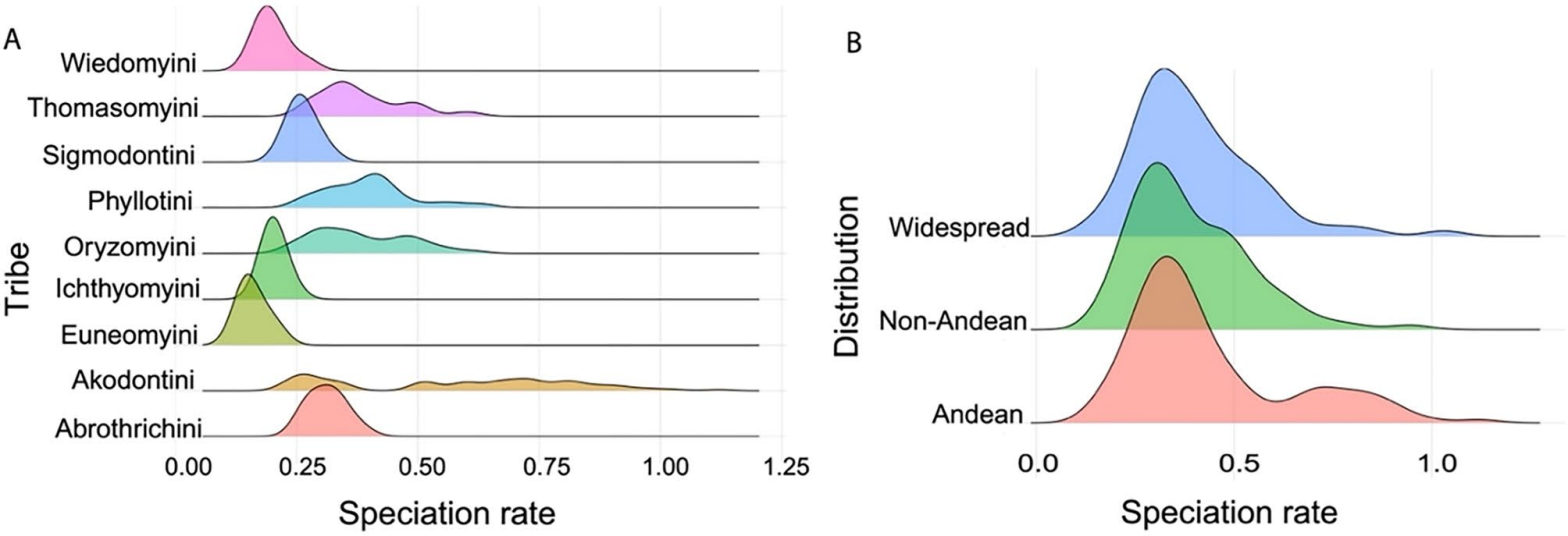
Tip speciation rates (387 values) under the ClaDS2 model (**A**) by tribe and (**B**) by distribution.

ClaDS and BAMM approaches show a similar diversification pattern, where sigmodontine rodents have an initially high rate of speciation that gradually decreases over time (Fig. 1 and Supplementary Information, Fig. S4). The diversification analyses performed with BAMM strongly rejected a constant-rate model (Bayes factor in Supplementary Information, Table S5). The best model configuration identified one shift in speciation rate in the speciose *Akodon* genus (Fig. 1B). ClaDS found that speciation rates ranged from 0.14 to 2.2 events per million years (Myr^-1^) showing variability in rates relatively high (Fig. 1A), as indicated by the σ value (σ = 0.45). Estimated hyperparameters confirm this general tendency for lower daughter rates than ancestral ones, as we can see from the trend parameter α (α = 0.756) and the mean relative change in speciation rate m = αxe^σ 2/2^ (m = 0.837). Due to that correspons to a ‘niche-filling’ scenario where diversification gets harder as new species arise [^91^; Supplementary Information, Fig. S5]. Also found a low level of extinction (ε = 0.034). When we split the estimated branch-specific speciation rates (772 total branches) by tribe, Euneomyini has the lower and homogeneous speciation rates in ranges from 0.05 to 0.08 Myr^−1^. While higher and heterogeneous speciation rates are in Akodontini (0.26–1.42 Myr^−1^) and Oryzomyini tribes (0.27–1.81 Myr^−1^; Fig. 1C). Moreover, for both methodological approaches, most tip speciation rates estimated (387 terminal branches) oscillate around 0.4 species per Myr, with higher speciation rates in the *Akodon* genus. However, ClaDS show a higher variability of estimated values across Sigmodontinae history than BAMM, which detected a unique and discrete shift rate (Supplementary Information, Fig. S6). Then, considering only the “tip-rates” by tribe, the Akodontini tribe presents higher speciation rates (Fig. 2A). Finally, the “tip-rates” separated by distribution regions show a similar trend between three areas (Andean, non-Andean, and widespread distribution). Yet, the Andean region presents a higher density of high speciation rates (Fig. 2B). Moreover, 80% of these higher speciation rates (> 0.7 species per Myr) are species of the *Akodon* genus with Andean distribution.

### Biogeographic history and dispersal rates

**Figure 3:**
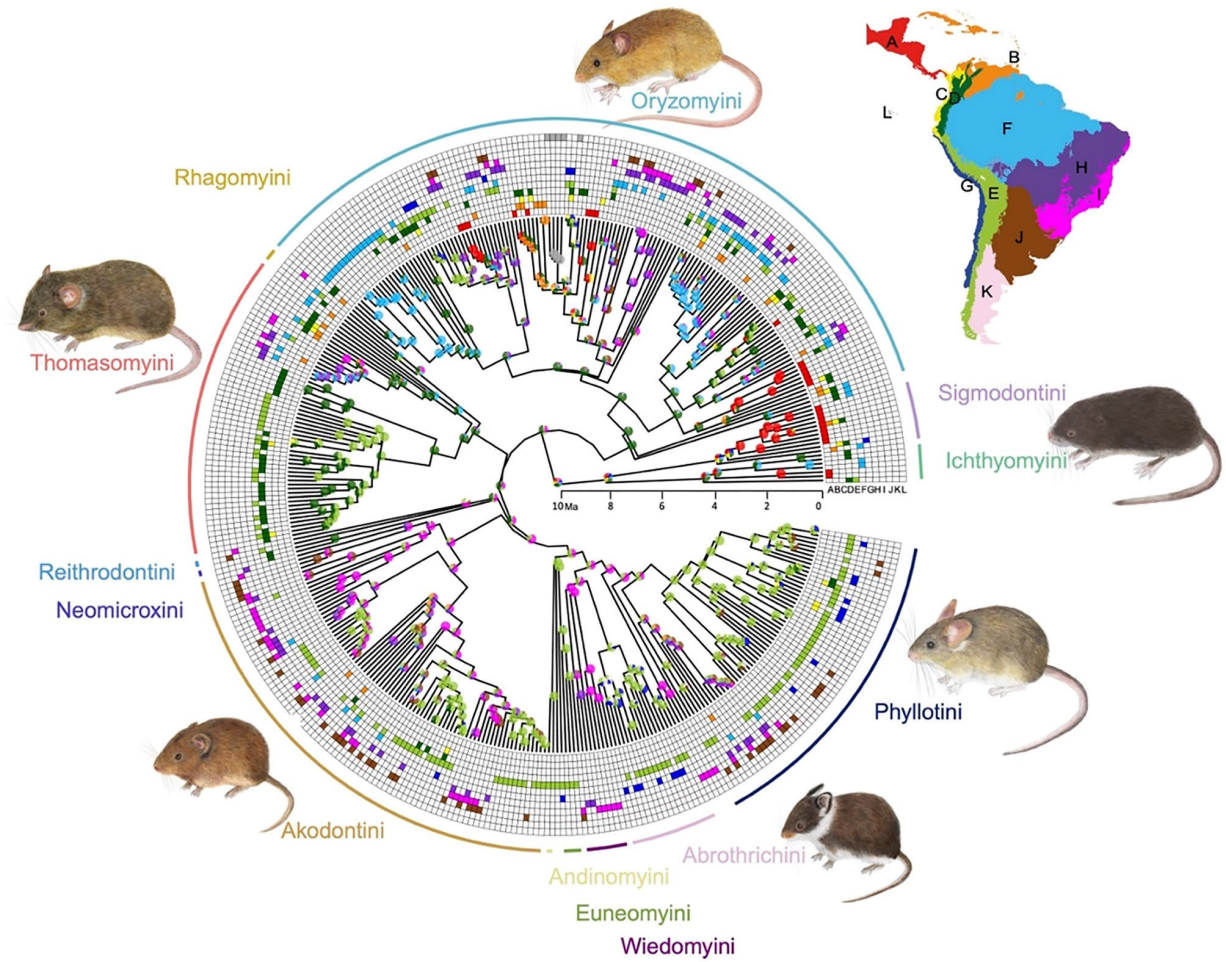
Ancestral range of sigmodontine rodents estimated under the Dispersal-Extinction-Cladogenesis model implemented in BioGeoBEARS. The relative probabilities of each biogeographic region are represented in pies at the nodes. The external boxes show the actual ranges of taxa, including Mesoamerica (**A**), Guinean savanna and Antilles Islands (**B**), Chocó and Tumbes (**C**), Northern Andes (**D**), Central and Southern Andes I, Amazonia (**F**), West South American coastal (**G**), Cerrado and Caatinga (**H**), Atlantic Forest (**I**), Chaco (**J**), Patagonian steppe (**K**), and Galápagos (**L**). Illustrations of rodents by Alayda Arce-Merma.

The best-fitted model was DEC (Supplementary Information, Table S7). Ancestral range estimations showed high uncertainty for the ancestral distribution of the most recent common ancestor (MRCA) of the Sigmodontinae subfamily, with the probabilities distributed among the different biogeographic regions (Northern Andes, Central-Southern Andes, and Amazonia). Sigmodontalia clade and Sigmodontini tribe show high uncertainty with high probabilities shared between Mesoamerica and Northern Andes (Fig. 3). Alternatively, the main tribes of the subfamily have Andean regions as the estimated ancestral range, supporting the idea that this area was the origin for the major clades in the subfamily (Fig. 3). Northern Andes is the most probable ancestral area for Oryzomalia (P = 0.84). The tribes that showed this ancestral area are Thomasomyini (P = 0.98), Neomicroxini (P = 0.97), Orizomyini (P = 0.98), Rhagomyini (P = 0.92), and Ichthyomyini (P = 0.58). Meanwhile, Central-Southern Andes is the most probable ancestral area for tribes Abrotrichini (P = 0.94), Andinomyini (P = 0.99), and Euneomyini (P = 0.99) (Fig. 3). Phyllotini has probabilities spread among Central and Southern Andes (P = 0.59) and Atlantic Forest (P = 0.57). On the other hand, the tribes Akodontini and Wiedomyini have an ancestral range estimated outside of The Andes, specifically the Atlantic Forest, as the most probable ancestral area (0.69 and 0.99, respectively) (Fig. 3).

**Figure 4:**
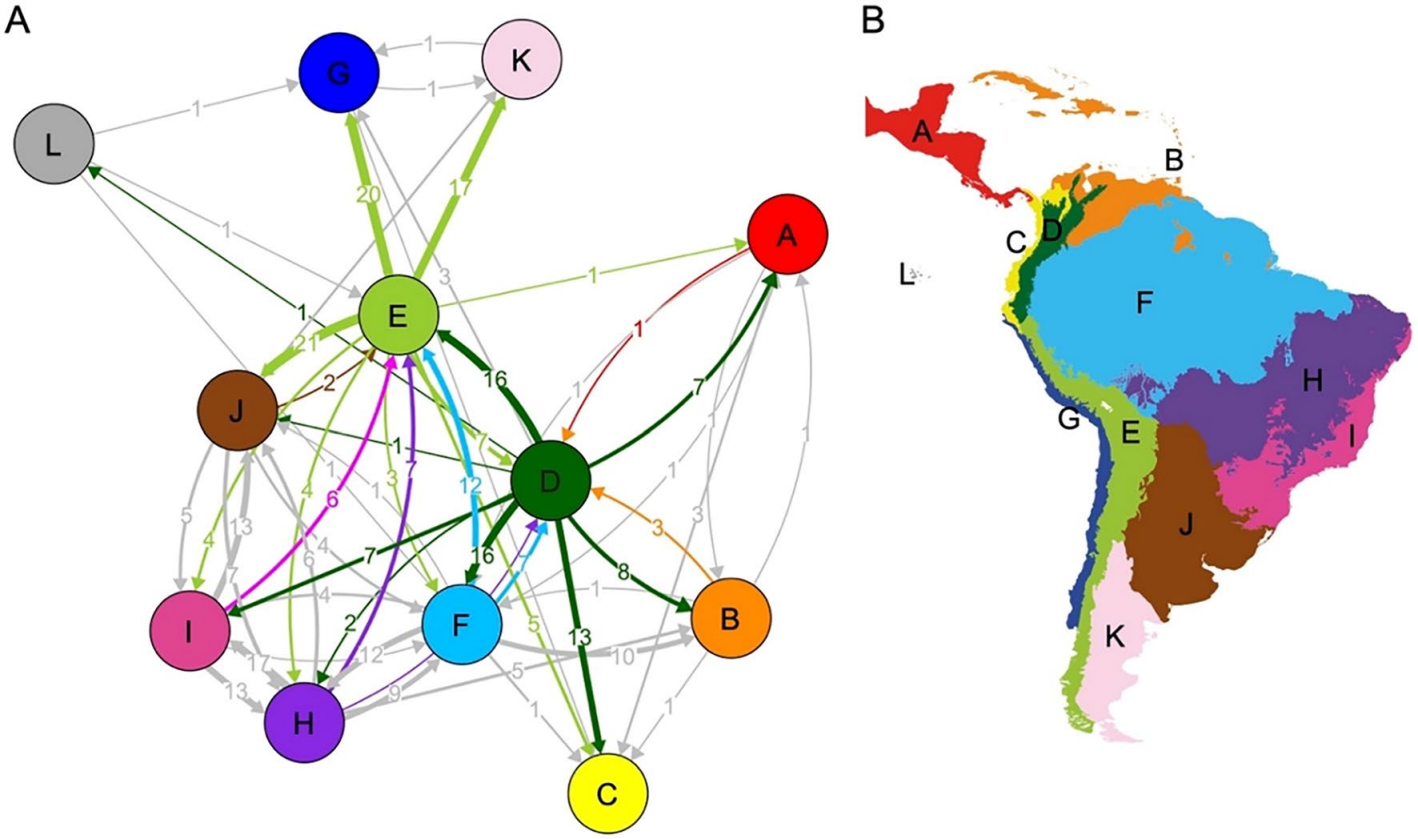
Estimated dispersal events among regions under the Dispersal-Extinction-Cladogenesis model implemented in BioGeoBEARS (**A**). Arrows indicate the direction and number of dispersal events, with line thickness proportional to the number of events. The position of the circles in the layout reflects the biotic connection among regions. Dispersal events out and into the Andes (Northern and Central-Southern) are highlighted in their respective color region (**B**). Interchange among non-Andean regions is shown in grey. See figure legend 3 for the names and detail of the biogeographic regions.

**Figure 5:**
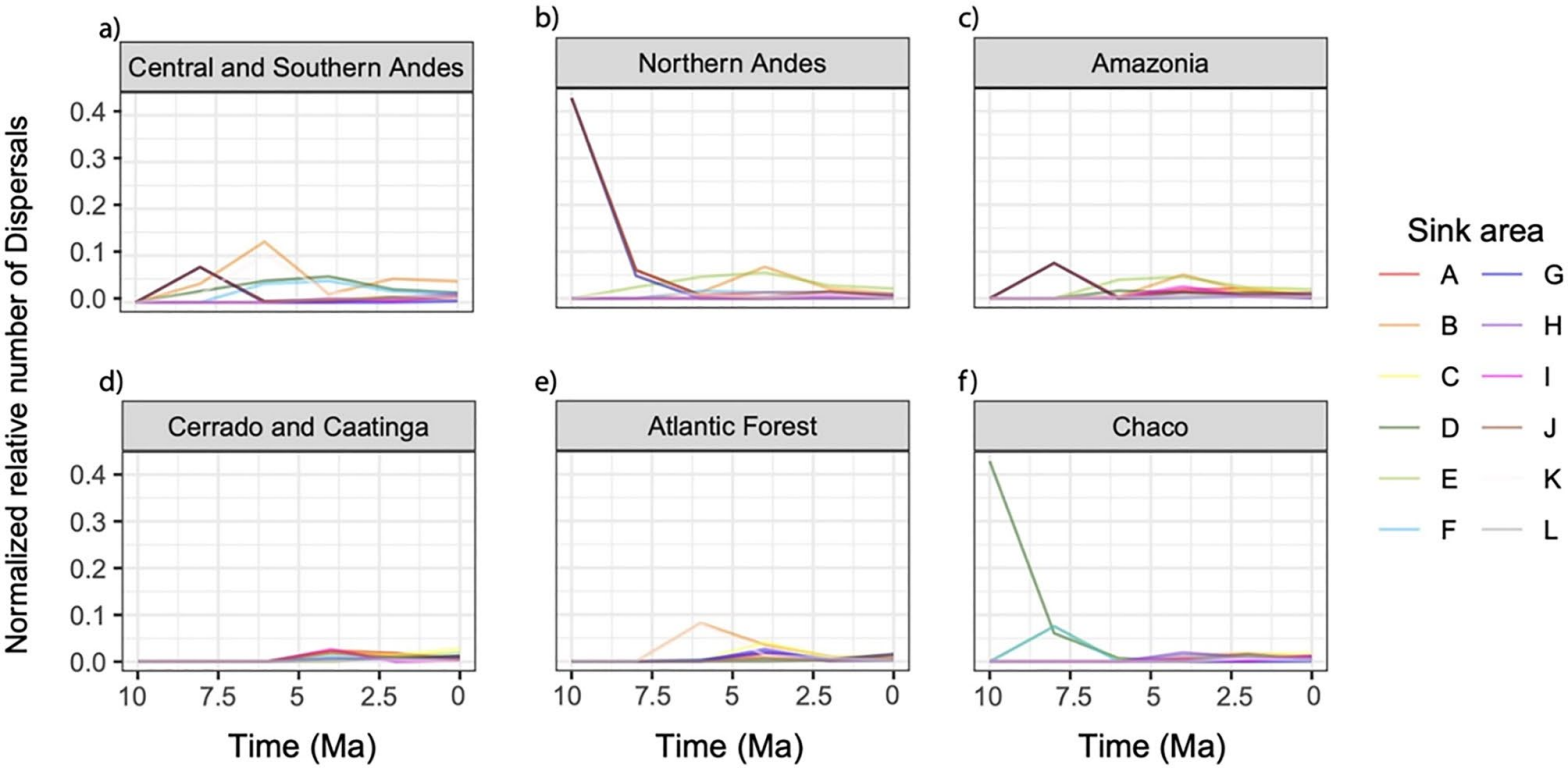
Colonization rates through time (relative number of dispersal events by dividing absolute numbers by the total length of all branches within each 2 Ma time bin) for the six main source regions (Table 1). Source regions (dispersal from) follow the colours in the key sink regions (dispersal to). See figure legend 3 for the names and detail of the biogeographic regions.

In addition, our biogeographic analyses show the Andean regions as the most important diversity sources for sigmodontine rodents, with 156 of 321 total estimated dispersal events (49% of all dispersal events) (Table 1). Thus, Central-Southern Andes (82) and Northern Andes (74) had about double the dispersal events than the most important sources of non-Andean regions, Amazonia (48) and Cerrado-Caatinga (45). Furthermore, the Central-Southern Andes was the most important sink, receiving the highest number of lineages (44; 14%), followed by Chaco (42), Cerrado and Caatinga (38), and Atlantic Forest (37) (Fig. 4, Table 1). The main source regions (Table 1) showed fluctuations in the relative number of dispersal events through time rather than constant rates (Fig. 5). Moreover, the number of relative dispersal events is concentrated at the beginning of the group diversification, during the Late Miocene and the Early Pliocene (10 to 5 Ma). These dispersal events mainly occurred (1) from Central and Southern Andes to Chaco and the Guinean savanna (Fig. 5a), (2) from Northern Andes to Mesoamerica, and West South American Costal (Fig. 5b), (3) from Chaco to Northern Andes and Amazonia (Fig. 5f).

**Table 1:** Regional sources and sinks of Sigmodontinae rodents’ diversity. Values correspond to dispersal events counted from the biogeographic analysis.

Region name	Source	Sink
Central and Southern Andes	82	44
Northern Andes	74	19
Amazonia	48	35
Cerrado and Caatinga	45	38
Atlantic Forest	33	37
Chaco	21	42
Guinean Savanna and Antilles Islands	6	24
Mesoamerica	6	10
Galápagos	3	1
West South American coastal	2	25
Patagonian Steppe	1	21
Chocó and Tumbes	0	25

### Andean uplift on diversification rates

We found an effect of the "Andean region" on the diversification rates. The best-fitting GeoHiSSE model had area-dependent speciation and one hidden trait (Model 13; Table 2). This "best model" had an AICc of 49.2 units lower than the next best-fitting model (Model 8), and an AIC weight of 0.999. All remaining models in the set had AIC weights below 0.0001. Here, the Andean region exhibited higher speciation rates than non-Andean regions. However, the extinction rate in Andean and non-Andean regions are almost identical (Table 3). These estimated values produce a higher net diversification rate (speciation–extinction) and turnover rate (speciation + extinction) in the Andean region compared to the non-Andean region (Table 3). Although geography has an important effect on diversification across the Sigmodontinae phylogeny, the diversification rates vary within each range as a function of some unobserved "hidden" trait. The higher turnover rates of clades in the Andean region occur without a hidden state associated (tau0A = 0.43), and the turnover rate declines when including a hidden state nested (tau0B = 0.14). Also, the non-Andean region shows a low turnover rate (tau1A = 2.06 × 10^–9^) and increases when including a hidden state nested (tau1B = 1.41). The ancestral range estimates the sigmodontines ancestor with higher node probability in widespread regions (0.67) vs. Andean and non-Andean regions (0.15 and 0.18, respectively).

**Table 2:** Estimated diversification rates models evaluated in the GeoHiSSE framework.

	Model		Range effect	Separate extirpation	Hidden states	No. Parameter	AIC	AICc	Model weight (wi)
Cladogenetic	1	CID-GeoSSE, only dispersal varies among areas	No	No	No	6	− 1088.946	2183.954	4.372e−32
	2	Canonical GeoSSE	Yes	No	No	7	− 1061.721	2133.599	3.941e−21
	3	CID-GeoHiSSE, 1 hidden trait	No	No	Yes	18	− 1074.364	2158.885	1.273e−26
	4	GeoHiSSE,1 hidden trait	Yes	No	Yes	20	− 1036.505	2095.713	8.735e−13
	5	GeoHiSSE, 1 hidden trait, full model	Yes	No	Yes	20	− 1035.745	2098.466	2.526e−13
Anagenetic	6	CID-anagenetic GeoSSE dispersal vary only	No	No	No	8	− 1045.210	2100.578	5.835e−14
	7	Anagenetic canonical GeoSSE	Yes	No	No	8	− 1043.123	2098.468	1.730e−13
	8	CID-anagenetic GeoHiSSE, 1 hidden trait	No	No	Yes	22	− 1037.710	2089.716	1.428e−11
	9	GeoHiSSE,1 hidden trait	Yes	No	Yes	22	− 1035.699	2089.875	1.444e−11
	10	GeoHiSSE, 1 hidden trait, full model	Yes	No	Yes	20	− 1035.747	2094.198	1.863e−12
Cladogenetic + Extirpation	11	Canonical GeoSSE + extirpation	Yes	Yes	No	8	− 1088.781	2189.783	2.567e−33
	12	CID-GeoHiSSE, 1 hidden trait + extirpation	No	Yes	Yes	20	− 1035.574	2093.852	2.214e−12
	*13*	*GeoHiSSE,1 hidden trait* + *extirpation*	*Yes*	*Yes*	*Yes*	*20*	− *1006.738*	*2040.452*	*0.999*

**Table 3:** Median value of parameters estimated from the average model.

Region	Speciation (s)	Extinction (x)	Net diversification (s–x)	Turnover (s + x)	Extinct fraction (x/s)
Andes	0.378	7.81e−10	0.378	0.378	2.06e−09
Non-Andes	0.273	5.65e−10	0.273	0.273	2.06e−09
Widespread	0.483	0	9.49e−01	1.27e−12	0

## Discussion

Our diversification and biogeographic processes analyses support the crucial role of the Andean mountains on the striking sigmodontine radiation. Most of the variation in net diversification and turnover rates through the sigmodontine radiation is linked to the Andes. The Northern and Central-Southern Andes were the most important source regions of diversity. At the same time, Central-Southern Andes is the most important sink of diversity, causing the Andean mountains to generate and receive the highest number of sigmodontine lineages throughout time. Therefore, the extant high Andean diversity results from macroevolutionary and biogeographical processes involving in situ speciation and several independent and recent immigrations. In this sense, we propose the Andes, as a "macroevolutionary cradle" and "species attractor" for sigmodontine lineages at different times in their evolutionary and biogeographical history. Moreover, Andean mountains may have had a "species-pump" effect to explain the subfamily's biogeographical history and current Neotropical distribution.

### Ancestral range estimation

Our results did not solve the uncertainty of the ancestral distribution of MRCA of the Sigmodontinae subfamily. Reig^60,61^ proposed an Andean origin for the subfamily in his seminal biogeographic study from 1986. However, several recent works suggest a sigmodontine origin in North America (Mexico) or Central America^57,65,66,104^. Nevertheless, all these efforts are only based on extant species. Ronez et al.^67^ proposed that if fossil lineages (*Copemys*, *Honeymys*) are considered Sigmodontinae relatives, sigmodontine's early history began in the north of Mexico. Interestingly, our results supported a Northern Andes origin for Thomasomyini and Oryzomyini tribes, agreeing with classical Reig^60,61^ proposal (although Reig included Thomasomyini inside the Oryzomyini tribe). However, this ancestral area disagrees with different recent proposals for oryzomyine origins: the Guiana Highlands/Amazon basin^58^, next to the Central Andes^62^, or the Boreal Brazilian region^105^. Regarding Thomasomyini, this tribe is a monophyletic group, with *Rhagomys* spp recognized as its sister clade at the tribal level [^77,106–108^]. Thomasomyini diversity is strongly concentrated in the Northern and Central Andes^68^. We estimated the ancestral range of the MRCA of Abrotrichini, Andinomyini, and Euneomyni tribes in Central-Southern Andes in agreement with previous proposals^58,62,65^. Also, our results support an origin in the Atlantic Forest for the MRCA of Akodontini (as well as Pardiñas et al.^109^ and Schenk and Steppan^58^) and Wiedomyini tribes. Our estimate of Phyllotini origin does not resolve the lack of consensus raised in previous works where their MRCA was estimated close and eastern to the Central Andes or out Andean Region^58,62,110,111^. The discrepancies observed between our ancestral area estimates and previous studies are due to at least two factors. First, differences in the delimitation of biogeographic regions used in the analysis. Maestri et al.^62^ assembled specific biogeographic areas for Sigmodontinae using the Infomap Bioregions clustering algorithm^112^. However, they argued that their biogeographic history could be inexact; the analyses might not localize the ancestral area given their large regions. Alternatively, we used biogeographic regions based on their geographic features, relatively abrupt habitat changes, and previous regionalization studies^58,70–72^. Second, the differences in the taxonomic sampling in the phylogeny and their respective species distribution. Here, we considered 387 of 489 sigmodontine recognized species. Thus, our analyses are based on datasets with considerably more taxonomic and updated sampling, especially for Andean groups previously poorly sampled (e.g., Thomasomyini)^58,62,107^.

### Macroevolutionary effect of the Andes

#### Cradle of diversity

The species richness pattern of sigmodontine diversity in the Andean mountains can be explained by the significant macroevolutionary influence of their striking orographic features. Here, we found that Andean mountains acted as a "cradle of diversity" for several sigmodontine groups, i.e., Andean lineages speciated faster than non-Andean lineages, leading to a rapid accumulation of species through time and with high turnover rates^14,15^. Biodiversity cradles represent areas of neoendemism. They are inhabited by recently diverged lineages, such as the radiation of plants in the high-altitude Andean Páramos or cichlids in the East African great lake^45,113,114^. Indeed, several nodes within the subfamily support the view that the Andes provided niche opportunities that ultimately triggered the significant radiation of several sigmodontine clades in the region, as the genus *Akodon* (39 species, 34 inhabiting the Andean Range and 21 Andean specialists^115^), the same node where Parada et al.^64^ found a significant increase using MEDUSA analysis.

Stebbins^15^ referred to cradles as geographical areas that present a particular set of characteristics that maximize the potential for diversification, expressed as the origination of biological novelties in terms of new species and traits. In cradles, the environmental conditions that most often trigger new radiations have two characteristics: they are unstable in time, meaning that the preferred habitat of a species shifts in space frequently, increasing chances of population fragmentation and reproductive isolation; and they are heterogeneous in space, meaning that external selective pressures that lead to population differentiation over time (e.g., differences in soil, temperature, and precipitation) are more diverse in a relatively small area. In other words, these areas increase the chances of triggering new radiations by both, leading to the endless opportunity for geographical isolation of populations and imposing external selective pressure to change.

For example, the valley systems located at equivalent elevations in the Peruvian Andes show radically different habitats. Some valleys are quite xeric and others very wet, offering horizontal and vertical differentiation opportunities for the *Akodon* genus^21,116,117^. Three species of climbing rats of the genus *Rhipidomys* occur in the main cordilleras of Colombia and Venezuela: *R. caucensis* in the Western, *R. latimanus* in the Central, and *R. fulviventer* in the Eastern Cordillera, apparently reflecting the vicariant effect of the Cauca and Magdalena valleys between these primary ranges^118^. Another taxonomic group that has shown a marked relationship between the geological history of the Andes and diversification processes is the Phyllotini tribe in the Altiplano^117^. Geographic variation studies in rice rats of the genus *Nephelomys*^119^ concluded that its geographic distribution agreed with proposed avian and anuran centers of endemism. The latter author hypothesizes *Nephelomys* diversification patterns to Andean uplift during the Neogene, one of the Andean autochthonous genera along with *Mindomys* and the recently described, *Pattonimus*, within the diverse Oryzomyini tribe^120^. Further, an important portion of oryzomyine’s remarkable diversity is associated with the Andean slopes of northern South America. Several authors using different methodologies identified these regions as major centers of oryzomyine species richness^60,61,68,73,121–124^. In addition, the most speciose sigmodontine genus, *Thomasomys*, is distributed strictly in the Andean range in premontane and montane forests and Paramo. Species of this genus inhabit shrubby and forested habitats from northern Venezuela to nearly 18°S in Cochabamba and Santa Cruz, southern Bolivia, including isolated ranges such as the Serranía de la Macarena in the same country. The elevational range for *Thomasomys* spans over 3300 m, from about 1200 m. a. s. l. to above 4500 m. a. s. l. and includes a series of species groups endemic to specific Andean formations^109,125,126^.

The Andean mountains would also be the cradle of the Abrotrichini, Andinomyini, and the recently described Neomicroxini tribe^62,108,127^. Abrotrichini, also known as the "Andean Clade," shows a marked basal split between fossorial and cursorial species associated with the vicariant process at the beginning of the formation of the Arid Diagonal as a result of the uplift of the Central Andes^127,128^. Abrotrichini is the most diverse tribe west of the southern Andes, where two of the most speciose and widely distributed tribes, Akodontini and Oryzomyini, are virtually absent^129^. On the other hand, Andinomyini is composed of two genera, *Andinomys* and *Punomys*, whose habitats include subtropical mountain forests to pre-Puna and semiarid Puna^130,131^. These two genera have not been found in sympatry. However, at several localities (e.g., Valle de La Paz, Bolivia), they occur in neighboring localities, although separated by elevation and habitat types: *Andinomys* prefers the semiarid portions of the valley up to 3600 m. a. s. l., whereas *Punomys* inhabits the barren, broken rock areas at elevations above 4500 m. a. s. l.^107,132,133^. Rodents of the Neomicroxini tribe are restricted and distributed along high-Andean (over 3000 m. a. s. l.) forests and shrubland-grassland Páramo habitats from Northern Andes^59^.

#### Species attractor

Extant diversity along the Andes chain can also be explained by the Andes acting as a "species attractor"^14,134,135^. This scenario of Andes as a sink of diversity suggests that geographical centers of extant diversity may not necessarily coincide with geographical centers of origin. The "species attractor" effect has been documented for the Andes, where mountain uplift created new habitats and favored the independent colonization of out-Andean groups^14,136^. In this sense, colonization and subsequent Andean diversification are also reported for *Thamnophlius* antshrikes^137^, *Pionus* parrots^138^, *Cyanolyca* jays^139^, hummingbirds^140^, and *Elaenia* flycatchers^141^. In the same way, the arrival of sigmodontine lineages originated outside the Andes was not the product of a single colonization event but, resulted from multiple independent colonization events by members of ramifying subclades. Our results show that the Central-Southern Andes was the most important sink, receiving the highest number of lineages (44; 14%). Many of these dispersal events to Andean mountains (both Northern and Central-Southern) occurred in the Late Miocene from the Chaco (Fig. 5a,f). They are supported by the congruent biogeographical patterns of several vertebrate groups that highlight even a possible past connection between the eastern (Atlantic Forest) and western (Andean mountains) communities, likely driven by Quaternary climatic changes^110,138,141,142^. Thus, the Andean mountains would have been colonized independently by members of different tribes, contributing to the co-occurrence of distantly related species^62^. Indeed, Northern Andes houses a remarkable diversity of oryzomyine genera. Most of these lineages are considered independent colonizers of the Andes (*Aegialomys*, *Mindomys-Pattonimus-Nephelomys*, *Handleyomys* (species of the "alfaroi group"; see^143^). As they belong to different clades within the tribe, they do not share recent common histories, suggesting that dispersal is the most important process of tribal diversification in the Andean mountains^58,62,120^. In the same way, Akodontini lineages colonized the Andes in several independent dispersal events giving rise to the Andean *Akodon* species, *Lenoxus*, and the Andean species of the genus *Oxymycterus*. Once in the Andes, several speciation events took place in situ, originating the extant diversity of this tribe in the Andes.

#### Species pump and turnover

Thus, the high diversity of this subfamily in the Andes would have resulted from in situ speciation (macroevolutionary cradle) and immigration (species attractor) from lowland areas. In addition, the Andes have also been the biogeographic source of multiple lineages to adjacent regions, acting as a "species pump"^16,64^. Northern Andes and the Central-Southern Andes are proposed as the most important diversity sources of sigmodontine lineages to the rest of the Neotropics from the Late Miocene until Pliocene (Fig. 5). These Andean regions had twice as many dispersal events as the most important non-Andean sources (see Table 1). With this, the Andes stand out as the center of early rodent diversification and diversity accumulation in the Neotropics^62,65^. For example, the phyllotine rodent *Calomys* contains two large clades. One clade (*C. musculinus*, *C. lepidus*, and *C. sorellus*) is distributed in the Andes with two species restricted to the Andean range (*C. lepidus* and *C. sorellus*), showing some local differentiation. In contrast, members of the second clade invaded the lowlands, including non-forested biomes, undergoing substantial radiations^117,144^. In line with our results, these examples show how that part of the ancient Andean diversity, once generated in situ, was essentially "pumped out" of the Andes.

The continuous origination of species in the Andes, whether by lineages that radiate in situ or arrive from an extra-Andean area, generates a high species turnover rate in the Andes^13,68^. Thus, the Andes would constitute a secondary contact zone between autochthonous and immigrant lineages that have been replaced over time by more recent lineages that co-occurred in this region, with topographic complexity that allows these to occupy diverse habitats zoned by elevation. The high elevational relief along both slopes of the Andes offers the best explanation for sigmodontine high turnover^68,145,146^. According to previous proposals, these results indicate that Central America, Amazonia, and Atlantic Forest show low turnover due to harbor assemblages of early diversifying and distantly related sigmodontine species^62,68^.

## Conclusions

Our results support the crucial role of the Andean Mountains in the sigmodontine radiation, acting as a "macroevolutionary cradle" and "species attractor" for several lineages at different times that explain the extant high Andean diversity of these rodents. Moreover, the Andes is hypothesized as a "species pump," becoming the biogeographic source of multiple lineages to the rest of the Neotropics. This contribution puts into perspective the importance of the continent's major mountainous formation as the primary geographic driver of the evolutionary and geographic history of Sigmodontinae. Considering the enormous specific and functional diversity of sigmodontine in the Neotropics, it is very likely that other notable geographic features of South America are also drivers of this diversity. Interestingly, these features are directly or indirectly linked to Andean orogenesis^40^.

## Supplementary Information

Supplementary Information 1.

Supplementary Table S1.

Supplementary Table S2.

Supplementary Table S4.

Supplementary Table S6.

Supplementary Table S8.

Supplementary Table S9.

Supplementary Table S10.

Supplementary Information 2.

Supplementary Information 3.

Supplementary Information 4.

Supplementary Information 5.

## Data Availability

No new data were generated for this study. The data used for this paper are available from the original sources cited in the Methods and Supplementary Information.
