# Supplementary material for: The importance of the Andes in the evolutionary radiation of Sigmodontinae (Rodentia, Cricetidae), the most diverse group of mammals in the Neotropics
Source: Sci Rep. 2023 Feb 7;13:2207. doi: 10.1038/s41598-023-28497-0 (PMC9905555; doi:10.1038/s41598-023-28497-0)
Supplement: Supplementary file 1 — Supplementary Information 1. [file 41598_2023_28497_MOESM1_ESM.docx]

SUPPLEMENTARY INFORMATION MATERIAL

**The importance of the Andes in the evolutionary radiation of Sigmodontinae (Rodentia, Cricetidae), the most diverse group of mammals in the Neotropics**

Paulo Vallejos-Garrido^1,3,6^, Kateryn Pino^1,3,5^, Oscar Inostroza-Michael^4^, Alexander Pari^1,3,5^, Nicolás Espinoza-Aravena^1,4^, Macarena Toledo-Muñoz^2,3^, Boris Castillo-Ravanal^2,3^, Viviana Romero-Alarcón^9^, Cristián E. Hernández^4,7^, R. Eduardo Palma^8^, Enrique Rodríguez-Serrano^3^

^1^*Programa de Doctorado en Sistemática y Biodiversidad, Facultad de Ciencias Naturales y Oceanográficas, Universidad de Concepción, Concepción, Chile;*

^2^*Programa de Magíster en Ciencias mención Zoología, Facultad de Ciencias Naturales y Oceanográficas, Universidad de Concepción, Concepción, Chile;*

^3^*Laboratorio de Mastozoología, Departamento de Zoología, Facultad de Ciencias Naturales y Oceanográficas, Universidad de Concepción, Concepción, Chile;*

^4^*Laboratorio de Ecología Evolutiva y Filoinformática, Departamento de Zoología, Facultad de Ciencias Naturales y Oceanográficas, Universidad de Concepción, Concepción, Chile;*

^5^*Museo de Historia Natural, Universidad Nacional de San Agustín de Arequipa, Perú;*

*^6^Vida Silvestre Investigadores Limitada, Chile;*

^7^*Universidad Católica de Santa María, Arequipa, Perú;*

^8^*Laboratorio de Biología Evolutiva, Departamento de Ecología, Facultad de Ciencias Biológicas, Pontificia Universidad Católica de Chile*

*^9^Universidad Católica de la Santísima Concepción*

Corresponding author: Enrique Rodríguez-Serrano: [enrodriguez@udec.cl](mailto:enrodriguez@udec.cl)

The supplementary materials include the biogeographic and molecular compiled data sets used in the analyses and the estimated phylogenetic trees given in separate files and additional results, figures, tables, and references that complement those in the main text.

**Supplementary results**

*Sigmodontinae phylogenetic hypothesis*

While a full discussion of the debate around the phylogenetic relationships of sigmodontines goes beyond the scope of this study, we describe here briefly the results of our phylogenetic analysis.

Our phylogenetic hypothesis analysis highly supports the monophyly of the Sigmodontinae subfamily and its main clades, Sigmodontalia and Oryzomyalia (BT = 100%, Fig. S1).

Moreover, phylogenetic relationships within and between main Oryzomyalia tribes present moderate to high bootstrap support (80% - 100%, Fig. S1). Such as Phyllotini (98%), Abrothrichini (90%), Akodontini (96%), Thomasomyini (83%), and Oryzomyini (97%). Furthermore, the Oryzomyini tribe was recovered as a sister tribe of a clade consisting of the remaining tribes (94%). The subsequent divergence event led to a clade with moderate support (83%), composed of Thomasomyini and the *insertae sedis* genera, *Chinchillula* and *Abrawayomys*, and a clade with the remaining oryzomalyd tribes. Within this remaining clade, we recovered two main clades, one composed of Reithrodontini and Akodontini tribes (86%), and the other includes a clade consisting of (Andinomyini, Neomicroxini) (83%) tribes as a sister clade of (Euneomyini (Wiedomyini, Abrothrichini) (Phyllotini, *Delomys* *insertae sedis* genus)) clade (85%).

The main phylogenetic relationships of sigmodontines obtained are highly congruent with Parada's et al. (2022) phylogenetic hypothesis, as we included their genomic data (UCEs) in our analysis. In addition, we considered the Wiedomyini tribe, the recently described Neomicroni and Rhagomyini [1,2], and the *insertae sedis* genera, *Chinchillulla*, *Abrawayomys,* and *Delomys*. Although, our matrix gene has considerable gaps with the inclusion of Parada's UCEs data. This genomic information helped obtain resolution within the Oryzomalya clade, something previously elusive in studies based on a few genes [e.g., 3,4,5]. Moreover, our approach resulted in a highly resolved and well-taxonomic sampling (389 of the 489 recognized species) phylogenetic hypothesis to sigmodontine rodents, compared to other well-sampling phylogenies taxonomy-based hypotheses [6].

**Supplementary tables**

**Table S1** (separate file). Digitalized occurrences of Sigmodontinae rodents.

**Table S2** (separate file). List of genes and taxon sampling used in the phylogenetic analyses.

**Table S3** (this document). List of fossil taxa used in the phylogenetic analysis of sigmodontines. The taxonomic assignments were use to restrict topological constraints in the FBD analysis based on the phylogenetic hypothesis obtained from the IQTREE phylo-analysis.

| Taxon | Stratigraphic provenance | Max-age | Min-age | Constraints for fossil placement | | Reference |
| --- | --- | --- | --- | --- | --- | --- |
|  |  |  |  | Tribe | Subfamily |  |
| *Neotoma sawrockensis* | Rexroad Fm. | 4.75 | 1.8 | - | Neotominae | Ronez et al. 2021 |
| *Tsaphanomys shotwelli* | Juniper Creek | 9.4 | 4.7 | - | Neotominae | Ronez et al. 2021 |
| *Repomys gusteylyi* | Horned Toad Fm. | 9.4 | 4.7 | - | Neotominae | Ronez et al. 2021 |
| *Honeymys esmeradelsis* | Claredonian USA (Nevada) | 13.6 | 10.3 | Stem | Sigmodontinae | Ronez et al. 2021 |
| *Honeymys mariae* | Claredonian USA (Nevada) | 13.6 | 10.3 | Stem | Sigmodontinae | Martin et al. 2020 |
| *Cholomys pearsoni* | Vorohué Fm. | 2.9 | 2.6 | - | Sigmodontinae | Reig 1980 |
| *Cordimus debuisonjei* | Highest Terrace, Curaçao | 2.3 | 1.3 | - | Sigmodontinae | Zijlstra et al. 2014 |
| *Cordimus raton* | Highest Terrace, Curaçao | 2.4 | 1.4 | - | Sigmodontinae | Zijlstra et al. 2014 |
| *"Akodon" kermacki* | Chapadmalal Fm. | 4 | 3 | Akodontini | Sigmodontinae | Ronez et al. 2021 |
| *Dankomys simpsoni* | Chapadmalal Fm. | 4 | 3 | Akodontini | Sigmodontinae | Reig 1978 |
| *Agathaeromys donovani* | Bonaire, Netherlands Antilles | 0.9 | 0.23 | Oryzomyini | Sigmodontinae | Zijlstra et al. 2010 |
| *Agathaeromys praeuniversitatis* | Bonaire, Netherlands Antilles | 0.9 | 0.23 | Oryzomyini | Sigmodontinae | Zijlstra et al. 2010 |
| *Carletonomys cailoi* | Ensenadan Fm. | 1.2 | 0.8 | Oryzomyini | Sigmodontinae | Pardiñas 2008 |
| *Dushimys larsi* | Duivelsklip, Curaçao | 1.5 | 1.4 | Oryzomyini | Sigmodontinae | Zijlstra et al. 2012 |
| *Reigomys primigenus* | Ensenadan Fm. | 1.2 | 0.8 | Oryzomyini | Sigmodontinae | Machado et al. 2013 |
| *Graomys dorae* | Chapadmalal Fm. | 4 | 3 | Phyllotini | Sigmodontinae | Ronez et al. 2021 |
| *Ichthyurodon ameghinoi* | San Andrés Fm. | 2.6 | 1.78 | Phyllotini | Sigmodontinae | Steppan & Pardiñas 1998 |
| *Kraglievichimys formosus* | Monte Hermoso Fm. | 6.8 | 4 | Phyllotini | Sigmodontinae | Ronez et al. 2021 |
| *Panchomys steppani* | Vorohué Fm. | 2.9 | 2.6 | Phyllotini | Sigmodontinae | Pardiñas 1997 |
| *Pardinamys humahuaquensis* | Uquía Fm. | 3 | 1.2 | Phyllotini | Sigmodontinae | Ortiz et al. 2012 |
| *Tafimys powelli* | Luján Fm. | 0.8 | 0.011 | Phyllotini | Sigmodontinae | Ortiz et al. 2000 |
| *Chukimys favaloroi* | Brochero Fm. | 5.3 | 2.6 | Reithrodontini | Sigmodontinae | Barbierè et al. 2016 |
| *Olympicomys vossi* | Vorohué & San Andrés Fms. | 2.9 | 1.78 | Reithrodontini | Sigmodontinae | Steppan & Pardiñas 1998 |
| *Reithrodon* sp. | Chapadmalal Fm. | 4 | 3 | Reithrodontini | Sigmodontinae | Ronez et al. 2021 |
| *Sigmodon hudspethensis* | Ft. Hancock & Love Fms. | 4.75 | 1.8 | Sigmodontini | Sigmodontinae | Ronez et al. 2021 |

**Table S4** (separate file). Sampling fractions of Sigmodontinae genera rodents for BAMM analysis.

**Table S5** (this document). Bayes Factor of diversification rate shifts as inferred from Sigmodontinae phylogeny.

|  |  | Denominator models | | | | | | | | |
| --- | --- | --- | --- | --- | --- | --- | --- | --- | --- | --- |
| Numerator models | Shifts | 0 | 1 | 2 | 3 | 4 | 5 | 6 | 7 | 8 |
|  | 0 | 1.000 | 0.167 | 0.244 | 0.476 | 1.024 | 2.698 | 7.170 | 9.731 | 17.029 |
|  | **1** | **5.981** | **1.000** | **1.460** | **2.846** | **6.126** | **16.134** | **42.882** | **58.196** | **101.844** |
|  | 2 | 4.095 | 0.685 | 1.000 | 1.949 | 4.195 | 11.047 | 29.362 | 39.848 | 69.734 |
|  | 3 | 2.101 | 0.351 | 0.513 | 1.000 | 2.152 | 5.668 | 15.066 | 20.446 | 35.781 |
|  | 4 | 0.976 | 0.163 | 0.238 | 0.465 | 1.000 | 2.634 | 7.000 | 9.500 | 16.625 |
|  | 5 | 0.371 | 0.062 | 0.091 | 0.176 | 0.380 | 1.000 | 2.658 | 3.607 | 6.313 |
|  | 6 | 0.139 | 0.023 | 0.034 | 0.066 | 0.143 | 0.376 | 1.000 | 1.357 | 2.375 |
|  | 7 | 0.103 | 0.017 | 0.025 | 0.049 | 0.105 | 0.277 | 0.737 | 1.000 | 1.750 |
|  | 8 | 0.059 | 0.010 | 0.014 | 0.028 | 0.060 | 0.158 | 0.421 | 0.571 | 1.000 |

**Table S6** (separate file). Presence-absence matrix used in BioGeoBEARS analysis.

**Tabla S7** (this document). Biogeographic model comparison tested in the BioGeoBEARS analysis, along with estimated parameters, log-likelihoods, and AIC values. The DEC model (in bold) is the best model.

|  | Log-Likelihood | N° parameters | *d* | *e* | *j* | AICc | AIC  weights |
| --- | --- | --- | --- | --- | --- | --- | --- |
| DEC | **-1279** | **2** | **0.044** | **0.019** | **0** | **2562** | **1** |
| DEC + *j* | -1375 | 3 | 0.02 | 4.40E-09 | 0.0095 | 2757 | 6.70E-43 |

**Table S8** (separate file). Presence-absence matrix used in GeoHiSSE analysis.

**Table S9** (separate file). Ancestral probabilities per region for each node of the Sigmodotinae phylogeny estimated in BioGeoBEARS analysis.

**Table S10** (separate file). Number of dispersal events between regions in BioGeoBEARS analysis.

**Supplementary figures:**

**
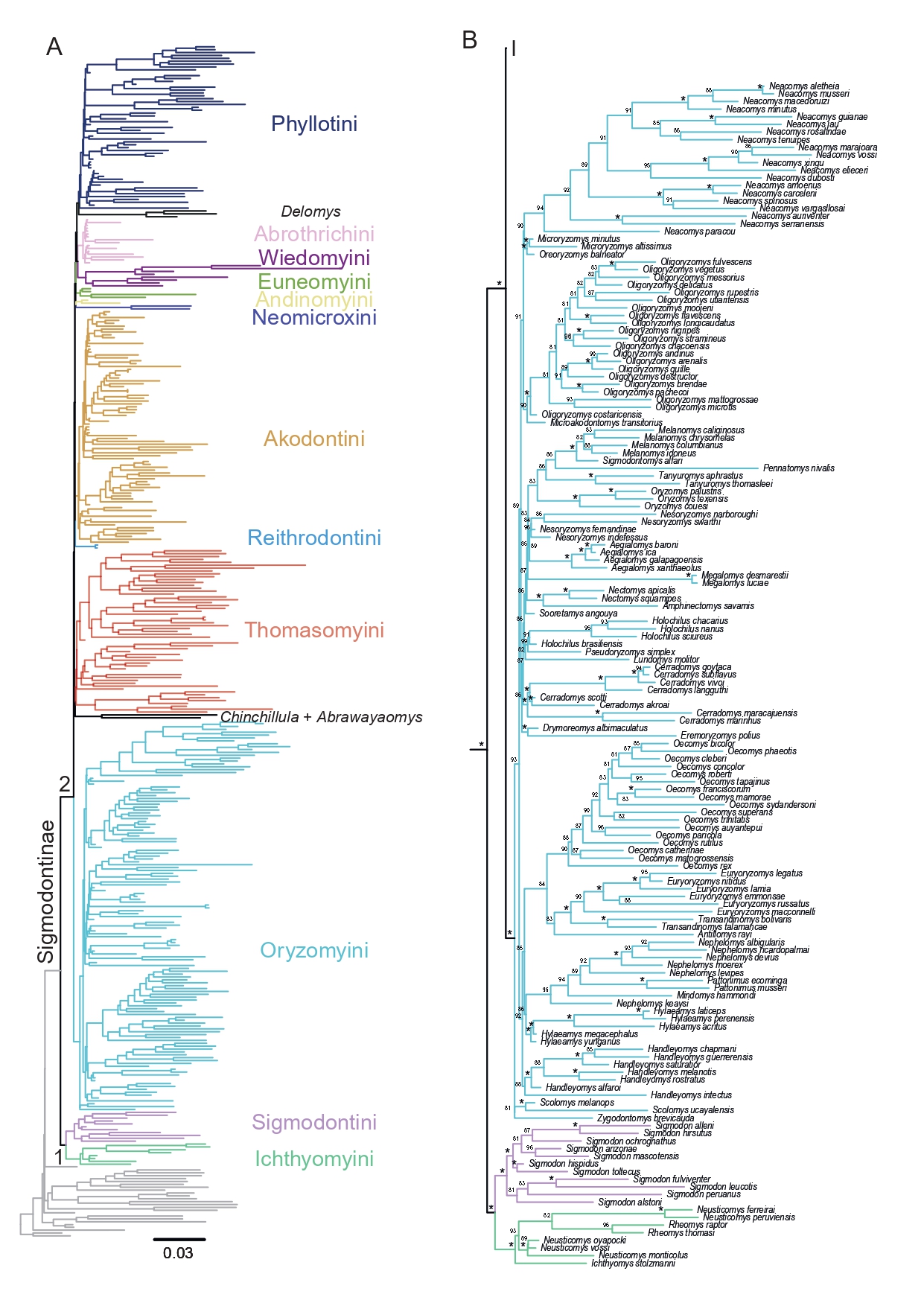
**

**
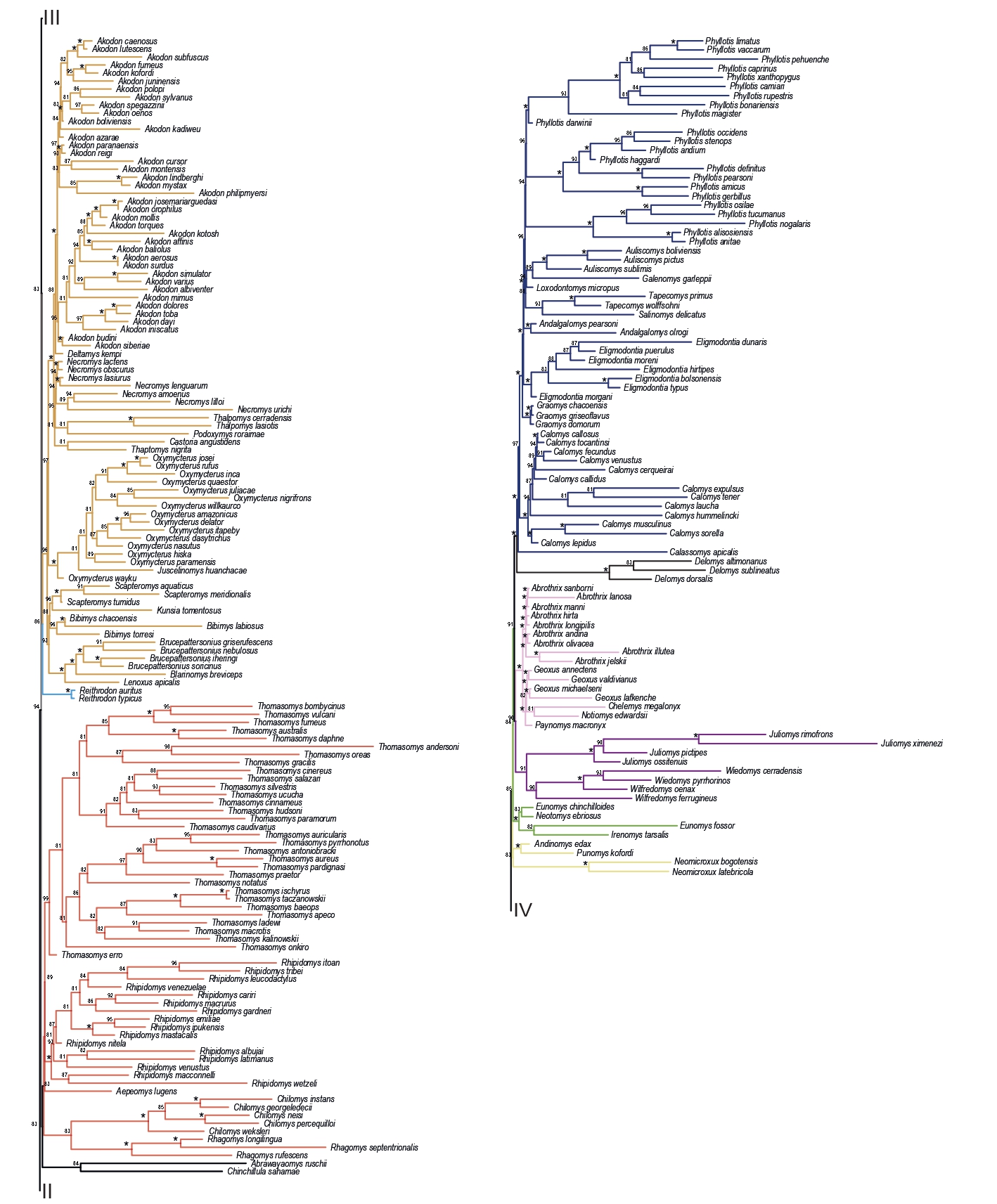
**

**Figure S1.** Maximum likelihood phylogenetic tree of the Sigmodontinae subfamily. (A) Complete phylogenetic tree colored by tribes, *incertae sedis* genera are indicated in black. The first divergence event leads to the main groups Sigmodontalia (1) and Oryzomyalia (2). Outgroup species are highlighted in grey. Scaled bar indicates the expected amount of change (number per substitution per site) along branches. (B) Phylogeny sectioned (I - IV) to show minor relationships within tribes. Numbers above branches are the ML bootstrap values; * indicates 98-100 %. The file for this phylogeny is available as **Supplementary Information File 4.**

**
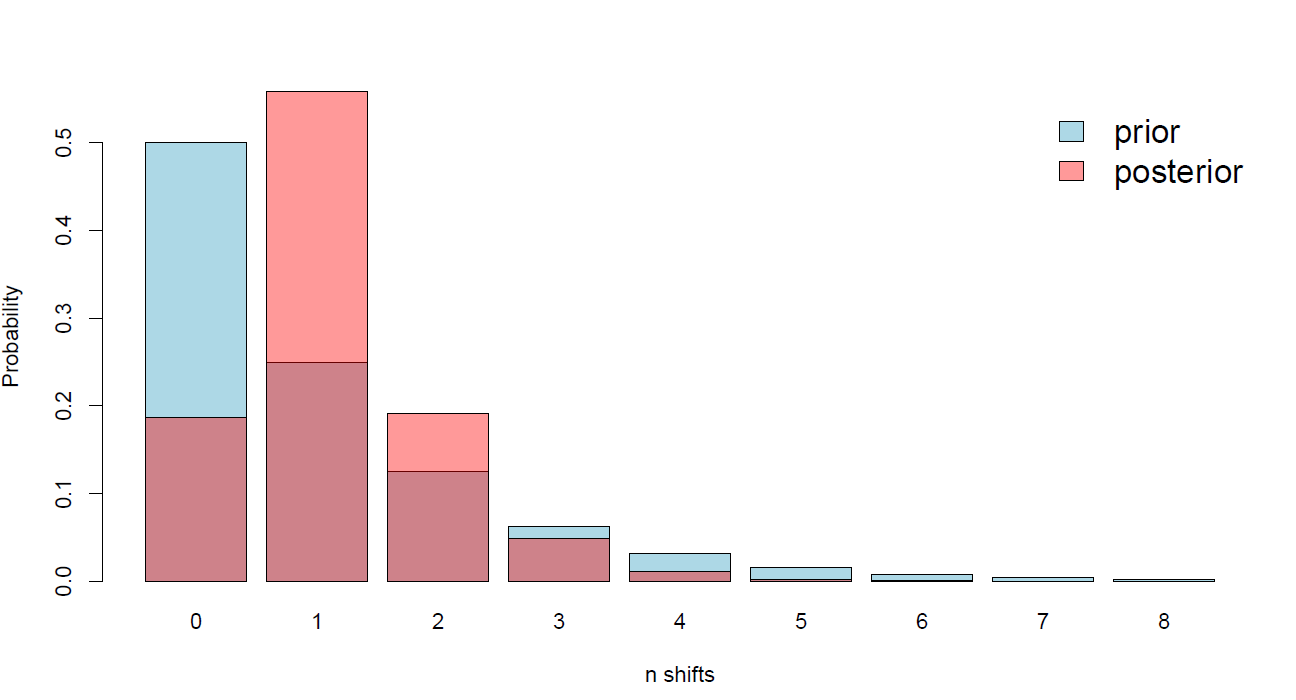
**

**Figure S2**. Prior and posterior distribution probabilities of the number of rate shifts estimated by BAMM.


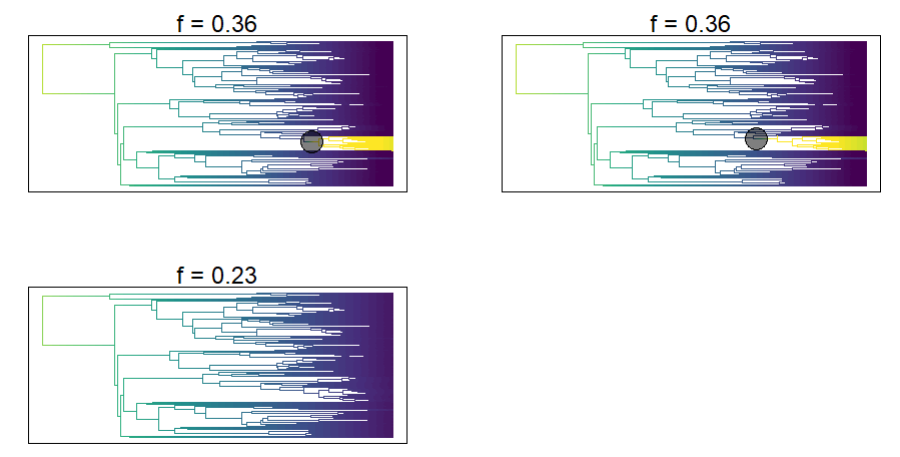


**Figure S3**. Macroevolutionary shift configurations and their frequencies show 95% credible set of shift configurations from BAMM analysis. Colour intensity across branches is proportional to changes in speciation rate from slower to faster rates (purple to yellow, respectively). Significant rate changes are pointed out with a circle along the branch at which this shift takes place. Note that among the 95% credible set of shift configurations, the majority (~36% of the shift configurations) indicate a significant increase in diversification rate at *Akodon* origin.


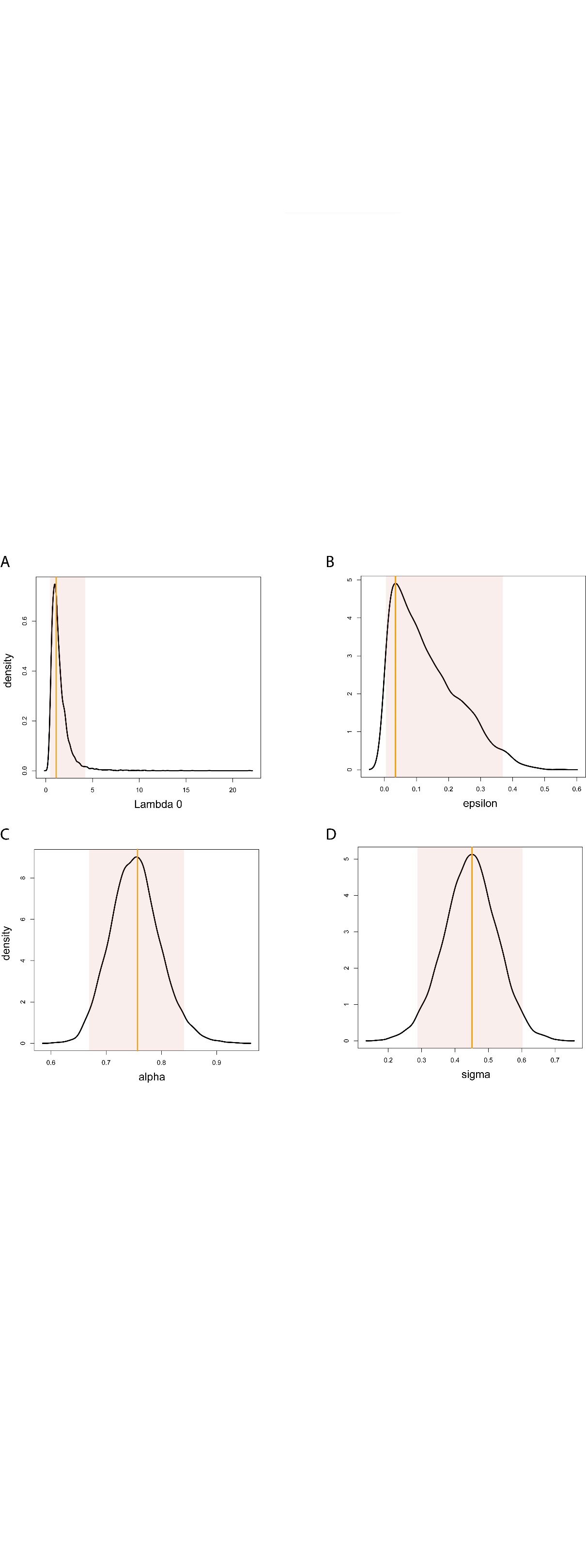


**Figure S4**. Plots of marginal posterior density of the parameters estimated by ClaDS. A) Lambda 0; B) epsilon; C) alpha; D) sigma.


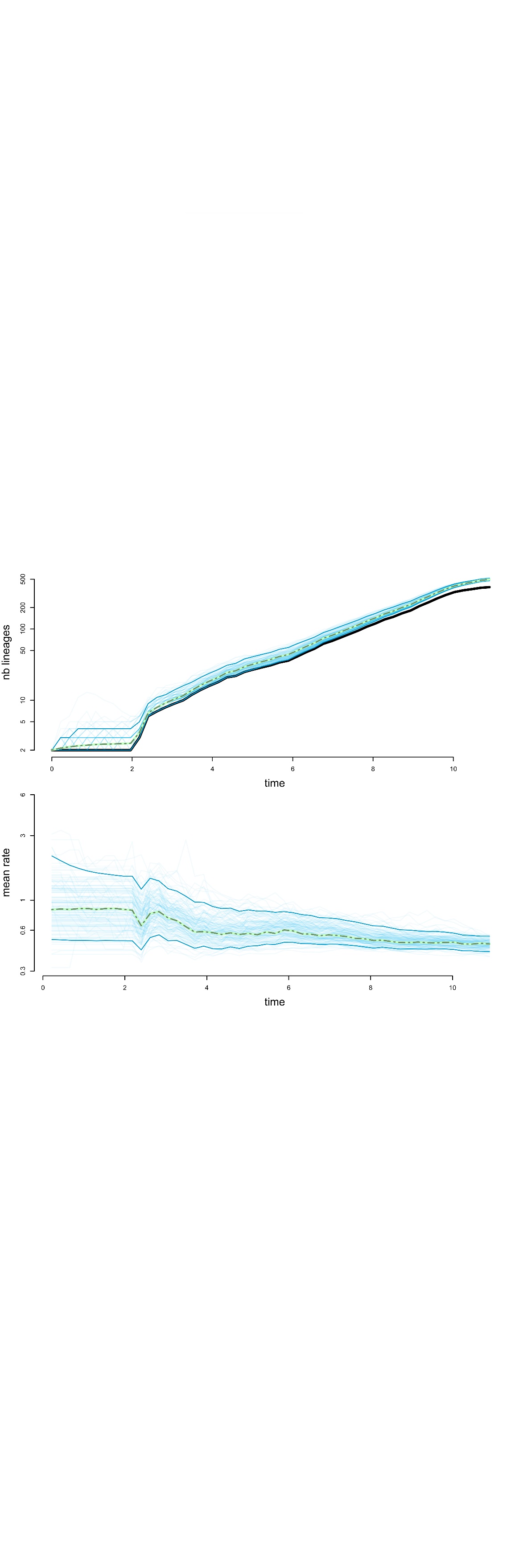


**Figure S5**. Above: DTT (number of lineages through time) plot obtained by ClaDS. Bottom: RTT (speciation rate through time) plot obtained by ClaDS. In both, the black line is the LTT plot (number of lineages through time in the reconstructed phylogeny). Thin blue lines: individual MCMC iterations. Thick blue lines represent the confidence interval, and dotted green lines are the point estimates.


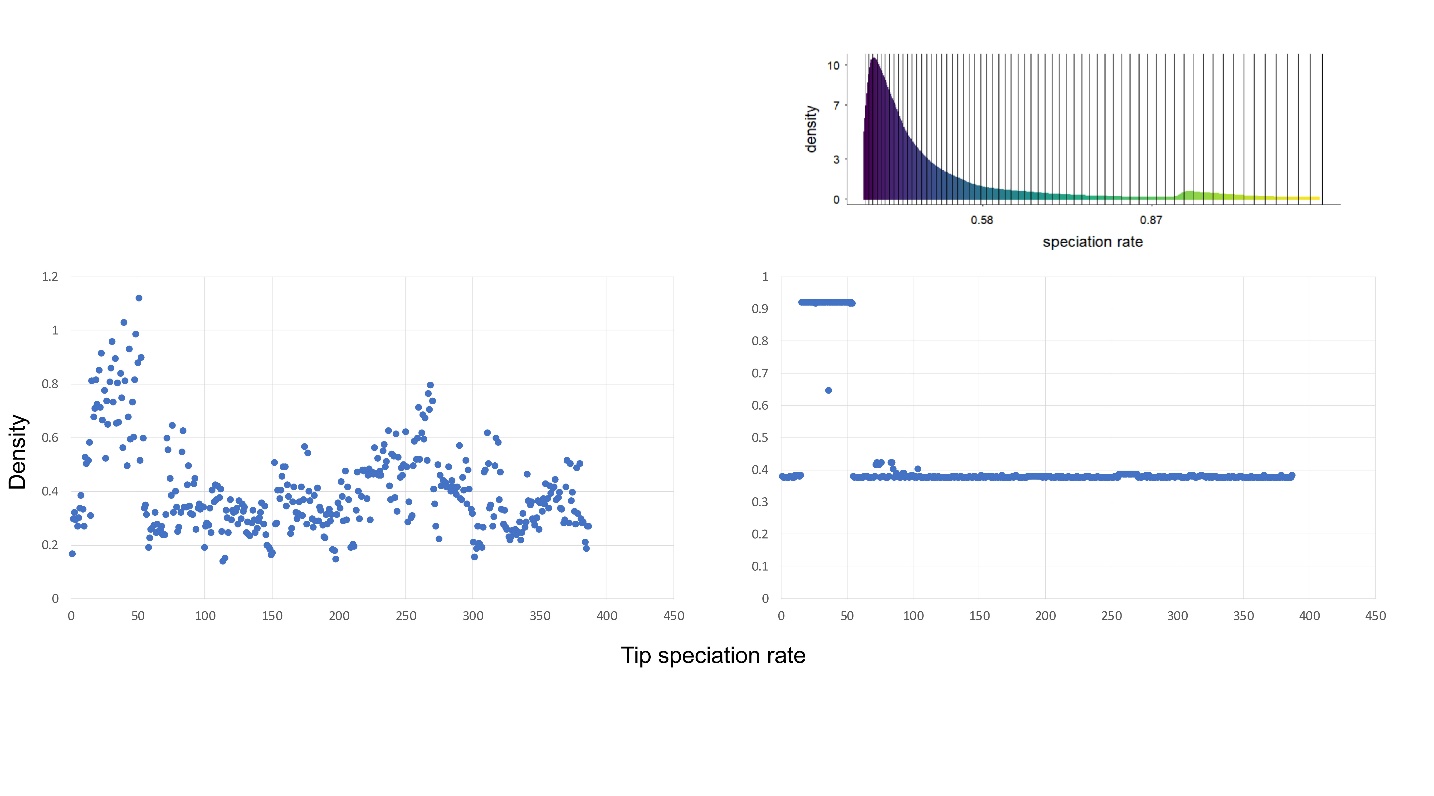


**Figure S6**. Left: Density distribution of tip speciation rate estimated by ClaDS model. Right: Density distribution of tip speciation rate estimated by BAMM model.


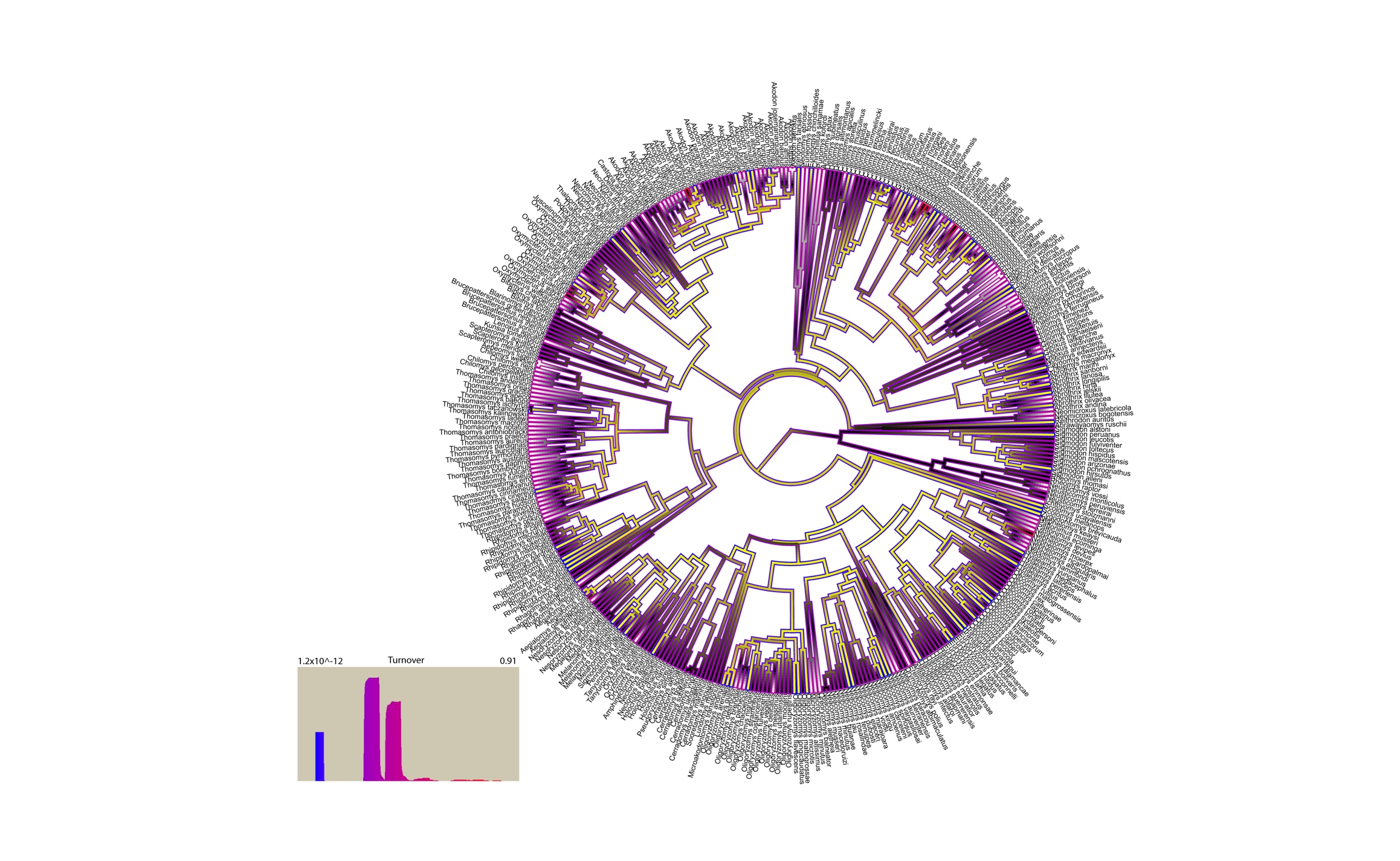


**Figure S7**. Geographic area reconstruction of areas and turnover rates across the set of 13 models. Lineages of the Andean region (white branches), non-Andean region (black branches), and widespread (yellow branches) across sigmodontine phylogeny. The branch border color ranges from the slowest turnover rates (blue) to the highest rates (red).

**
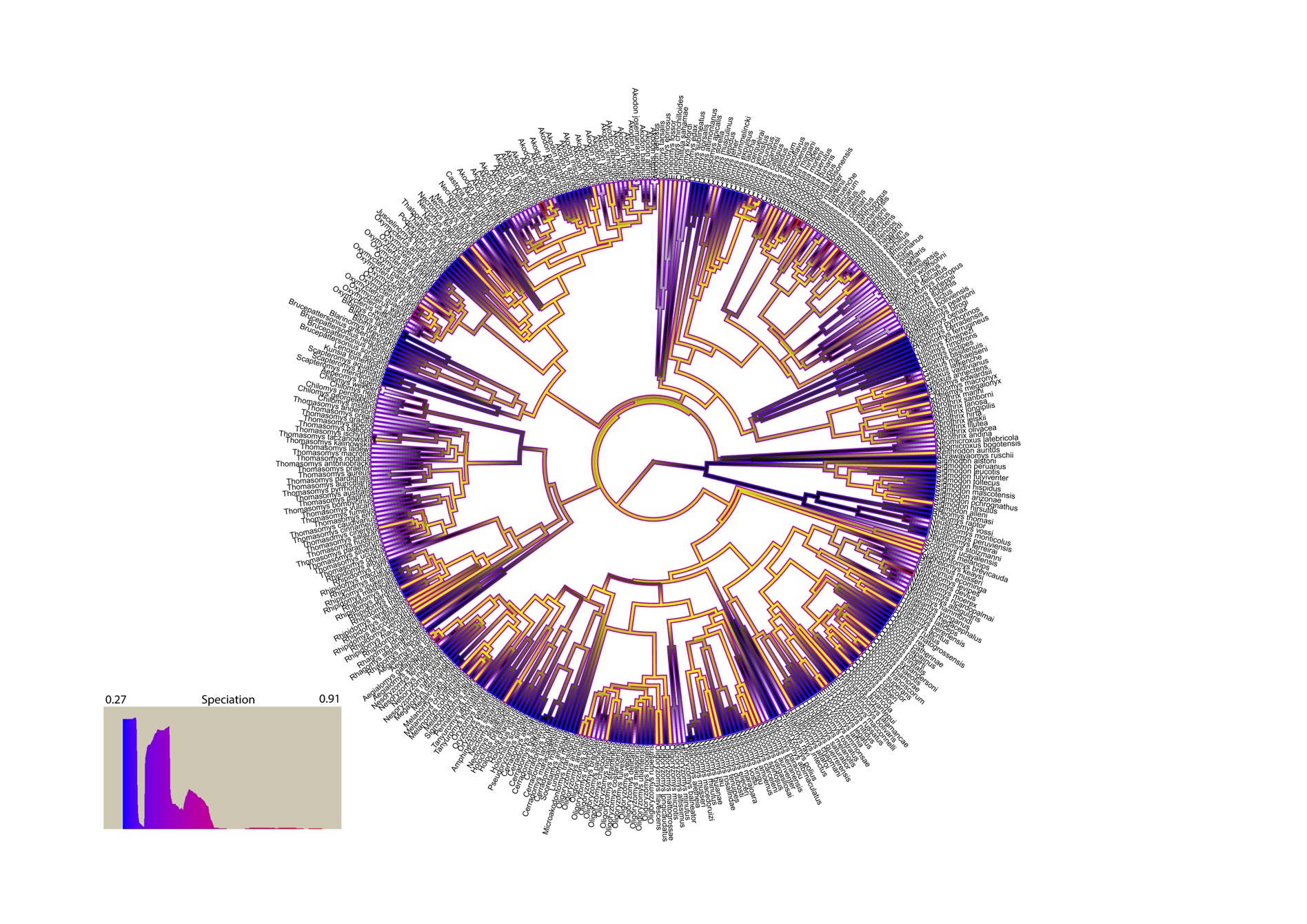
**

**Figure S8.**  Geographic area reconstruction of areas and speciation rates across the set of 13 models. Lineages of the Andean region (white branches), non-Andean region (black branches), and widespread (yellow branches) across sigmodontine phylogeny. The branch border color ranges from the slowest speciation rates (blue) to the highest rates (red).

**Supporting References**

**List of references cited in supporting results seccion.**

1. Pardiñas, U. F. J., Curay, J., Brito, J. & Cañón, C. A unique cricetid experiment in the northern high-Andean Páramos deserves tribal recognition. *Journal of Mammalogy* **102**, 155–172 (2021).

2. Pardiñas, U. F. J. *et al.* Morphological disparity in a hyperdiverse mammal clade: a new morphotype and tribe of Neotropical cricetids. *Zoological Journal of the Linnean Society* **196**, 1013–1038 (2022).

3. Gonçalves, P. R. *et al.* Unraveling Deep Branches of the Sigmodontinae Tree (Rodentia: Cricetidae) in Eastern South America. *J Mammal Evol* **27**, 139–160 (2020).

4. Maestri, R. et al. The ecology of a continental evolutionary radiation: Is the radiation of sigmodontine rodents adaptive? *Evolution* **71**, 610–632 (2017).

5. Parada, A., Pardiñas, U. F. J., Salazar-Bravo, J., D’Elía, G. & Palma, R. E. Dating an impressive Neotropical radiation: Molecular time estimates for the Sigmodontinae (Rodentia) provide insights into its historical biogeography. *Molecular Phylogenetics and Evolution* **66**, 960–968 (2013).

6. Upham, N. S., Esselstyn, J. A. & Jetz, W. Inferring the mammal tree: Species-level sets of phylogenies for questions in ecology, evolution, and conservation. *PLoS Biol* **17**, e3000494 (2019).

**List of references revised to compile the multigene matrix for phylogenetic analyses.**

1. Abreu-Júnior, E. F. de & Percequillo, A. R. Small mammals of the Estação Ecológica de Bananal, southeastern Atlantic Forest, Brazil, with description of a new species of *Brucepattersonius* (Rodentia, Sigmodontinae). *Arq. Zool.* **50**, 1–116 (2019).
2. Abreu, E. F. *et al.* Systematics of *Brucepattersonius* Hershkovitz, 1998 (Rodentia, Sigmodontinae): molecular species delimitation and morphological analyses suggest an overestimation in species diversity. *Systematics and Biodiversity* **19**, 544–570 (2021).
3. Brace, S., Turvey, S. T., Weksler, M., Hoogland, M. L. P. & Barnes, I. Unexpected evolutionary diversity in a recently extinct Caribbean mammal radiation. *Proceedings of the Royal Society B: Biological Sciences* **282**, 20142371 (2015).
4. Brito, J. *et al.* A new genus of oryzomyine rodents (Cricetidae, Sigmodontinae) with three new species from montane cloud forests, western Andean cordillera of Colombia and Ecuador. *PeerJ* **8**, e10247 (2020).
5. Brito, J. *et al.* Unlocking Andean sigmodontine diversity: five new species of *Chilomys* (Rodentia: Cricetidae) from the montane forests of Ecuador. *PeerJ* **10**, e13211 (2022).
6. Brito, J. M. *et al.* New species of arboreal rat of the genus *Rhipidomys* (Cricetidae, Sigmodontinae) from Sangay National Park, Ecuador. *Neotropical Biodiversity* **3**, 65–79 (2017).
7. Cañón, C., Mir, D., Pardiñas, U. F. J., Lessa, E. P. & D'Elía, G. A multilocus perspective on the phylogenetic relationships and diversification of rodents of the tribe Abrotrichini (Cricetidae: Sigmodontinae). *Zool Scr* **43**, 443–454 (2014).
8. Christoff, A. U. *et al.* A new species of *Juliomys* (Rodentia, Cricetidae, Sigmodontinae) from the Atlantic Forest of Southern Brazil. *Journal of Mammalogy* **97**, 1469–1482 (2016).
9. Colmenares-Pinzón, J. E. Calling for a reassessment of rodent diversity in Colombia: description of a new species of *Neacomys* (Cricetidae: Oryzomyini) from the Magdalena Valley, with a new phylogenetic hypothesis for the genus and comments on its diversification. *Zootaxa* **4920**, 451–494 (2021).
10. Coyner, B. S., Braun, J. K., Mares, M. A. & Van Den Bussche, R. A. Taxonomic validity of species groups in the genus *Akodon* (Rodentia, Cricetidae). *Zool Scr* **42**, 335–350 (2013).
11. D'Elía, G. Phylogenetics of Sigmodontinae (Rodentia, Muroidea, Cricetidae), with special reference to the akodont group, and with additional comments on historical biogeography. *Cladistics* **19**, 307–323 (2003).
12. D'Elía, G., Hanson, J. D., Mauldin, M. R., Teta, P. & Pardiñas, U. F. J. Molecular systematics of South American marsh rats of the genus *Holochilus* (Muroidea, Cricetidae, Sigmodontinae). *Journal of Mammalogy* **96**, 1081–1094 (2015).
13. Di-Nizo, C. B., Suárez-Villota, E. Y. & Silva, M. J. J. Species limits and recent diversification of *Cerradomys* (Sigmodontinae: Oryzomyini) during the Pleistocene. *PeerJ* **10**, e13011 (2022).
14. Fernandes Semedo, T. B. *et al.* Systematics of Neotropical Spiny Mice, Genus *Neacomys* Thomas, 1900 (Rodentia: Cricetidae), from Southeastern Amazonia, with Descriptions of Three New Species. *American Museum Novitates* **2020**, 1 (2020).
15. Gonçalves, P. R. *et al.* Unraveling Deep Branches of the Sigmodontinae Tree (Rodentia: Cricetidae) in Eastern South America. *J Mammal Evol* **27**, 139–160 (2020).
16. Guilardi, M. D. *et al.* A review of *Euryoryzomys legatus* (Rodentia, Sigmodontinae): morphological redescription, cytogenetics, and molecular phylogeny. *PeerJ* **8**, e9884 (2020).
17. Hanson, J. D. *et al.* A new species of fish-eating rat, genus *Neusticomys* (Sigmodontinae), from Ecuador. *Zool. Stud.* **54**, 49 (2015).
18. Hanson, J. D. Molecular phylogenetics of Oryzomyini: does a multigene approach help resolve a systematic conundrum? (2008).
19. Hanson, J. D., Indorf, J. L., Swier, V. J. & Bradley, R. D. Molecular divergence within the *Oryzomys palustris* complex: evidence for multiple species. *Journal of Mammalogy* **91**, 336–347 (2010).
20. Hurtado, N. & D'Elía, G. An assessment of species limits of the South American mouse genus *Oligoryzomys* (Rodentia, Cricetidae) using unilocus delimitation methods. *Zool Scr* **48**, 557–570 (2019).
21. Hurtado, N. & D'Elía, G. Historical biogeography of a rapid and geographically wide diversification in Neotropical mammals. *Journal of Biogeography* **49**, 781–793 (2022).
22. Hurtado, N. & Pacheco, V. Revision of *Neacomys spinosus* (Thomas, 1882) (Rodentia: Cricetidae) with emphasis on Peruvian populations and the description of a new species. *Zootaxa* **4242**, 401 (2017).
23. Jayat, J. P. *et al.* The *Phyllotis xanthopygus* complex (Rodentia, Cricetidae) in central Andes, systematics and description of a new species. *Zool Scr* **50**, 689–706 (2021).
24. Jayat, J. P., D’Elía, G., Ortiz, P. E. & Teta, P. A new species of the rodent genus *Necromys* Ameghino (Cricetidae: Sigmodontinae: Akodontini) from the Chaco Serrano grasslands of northwestern Argentina. *Journal of Mammalogy* **97**, 1321–1335 (2016).
25. Jayat, J. P., D’Elía, G., Pardiñas, U. F. J., Miotti, M. D. & Ortiz, P. E. A new species of the genus *Oxymycterus* (Mammalia: Rodentia: Cricetidae) from the vanishing Yungas of Argentina. *Zootaxa* **1911**, 31–51 (2008).
26. Machado, L. F. *et al.* Molecular phylogenetic position of endangered *Wilfredomys* within Sigmodontinae (Cricetidae) based on mitochondrial and nuclear DNA sequences and comments on Wiedomyini. *Zootaxa* **3986**, 421 (2015).
27. Martínez, J. J. & Gardenal, C. N. Phylogenetic relationships among species of the Neotropical genus *Graomys* (Rodentia: Cricetidae): contrasting patterns of skull morphometric variation and genetic divergence. *Biol. J. Linn. Soc.* **118**, 648–667 (2016).
28. Ojeda, A. A. *et al.* DNA barcodes highlight genetic diversity patterns in rodents from lowland desert and Andean areas in Argentina. *Molecular Ecology Resources* **22**, 2349–2362 (2022).
29. Ojeda, A. A. *et al.* Phylogenetic relationships among cryptic species of the *Phyllotis xanthopygus* complex (Rodentia, Cricetidae). *Zool Scr* **50**, 269–281 (2021).
30. Pacheco, V. *et al.* A revision of *Neusticomys peruviensis* (Rodentia: Cricetidae) with the description of a new subspecies. *Journal of Mammalogy* **101**, 858–871 (2020).
31. Pardiñas, U. F. J. *et al.* Gross stomach morphology in akodontine rodents (Cricetidae: Sigmodontinae: Akodontini): a reappraisal of its significance in a phylogenetic context. *Journal of Mammalogy* **101**, 835–857 (2020).
32. Pardiñas, U. F. J., Curay, J., Brito, J. & Cañón, C. A unique cricetid experiment in the northern high-Andean Páramos deserves tribal recognition. *Journal of Mammalogy* **102**, 155–172 (2021).
33. Pardiñas, U. F. J., Teta, P., Salazar-Bravo, J., Myers, P. & Galliari, C. A. A new species of arboreal rat, genus *Oecomys* (Rodentia, Cricetidae) from Chaco. *Journal of Mammalogy* **97**, 1177–1196 (2016).
34. Peçanha, W. T. *et al.* A new species of Oxymycterus (Rodentia: Cricetidae: Sigmodontinae) from a transitional area of Cerrado – Atlantic Forest in southeastern Brazil. *Journal of Mammalogy* **100**, 578–598 (2019).
35. Percequillo, A. R., Weksler, M. & Costa, L. P. A new genus and species of rodent from the Brazilian Atlantic Forest (Rodentia: Cricetidae: Sigmodontinae: Oryzomyini), with comments on oryzomyine biogeography: Oryzomyini phylogeny and biogeography. *Zoological Journal of the Linnean Society* **161**, 357–390 (2011).
36. Pine, R. H., Timm, R. M. & Weksler, M. A newly recognized clade of trans-Andean Oryzomyini (Rodentia: Cricetidae), with description of a new genus. *Journal of Mammalogy* **93**, 851–870 (2012).
37. Pinotti, J. D. *et al.* Multiple refugia and glacial expansions in the Tucumane–Bolivian Yungas: The phylogeography and potential distribution modeling of *Calomys fecundus* (Thomas, 1926) (Rodentia: Cricetidae). *J. Zool. Syst. Evol. Res.* **58**, 1359–1373 (2020).
38. Prado, J. R. do, Knowles, L. L. & Percequillo, A. R. New species boundaries and the diversification history of marsh rat taxa clarify historical connections among ecologically and geographically distinct wetlands of South America. *Molecular Phylogenetics and Evolution* **155**, 106992 (2021).
39. Rengifo, E. M. & Pacheco, V. M. Taxonomic revision of the Andean leaf-eared mouse, *Phyllotis andium* Thomas 1912 (Rodentia: Cricetidae), with the description of a new species. *Zootaxa* **4018**, 349 (2015).
40. Rengifo, E. M. & Pacheco, V. Phylogenetic position of the Ancash leaf-eared mouse *Phyllotis definitus* Osgood 1915 (Rodentia: Cricetidae). *Mammalia* **82**, 153–166 (2018).
41. Rocha, R. G. *et al.* Cryptic diversity in the *Oecomys roberti* complex: revalidation of *Oecomys tapajinus* (Rodentia, Cricetidae). *Journal of Mammalogy* **99**, 174–186 (2018).
42. Ruelas, D. & Pacheco, V. Taxonomic status and phylogenetic position of *Oxymycterus juliacae* Allen 1900 (Rodentia: Cricetidae). *Mammalia* **86**, 196–214 (2022).
43. Ruelas, D., Pacheco, V. & Jiménez, C. F. Range extension and phylogenetic relationships of *Akodon josemariarguedasi* (Rodentia: Cricetidae) with comments on the *aerosus* species group. *Mammalia* **84**, 207–213 (2020).
44. Ruelas, D., Pacheco, V., Inche, B. & Tinoco, N. A preliminary review of *Nephelomys albigularis* (Tomes, 1860) (Rodentia: Cricetidae), with the description of a new species from the Peruvian montane forests. *Zootaxa* **5027**, 175–210 (2021).
45. Salazar-Bravo, J., Pardiñas, U. F. J. & D'Elía, G. A phylogenetic appraisal of Sigmodontinae (Rodentia, Cricetidae) with emphasis on phyllotine genera: systematics and biogeography. *Zoologica Scripta* **42**, 250–261 (2013).
46. Saldanha, J. & Rossi, R. V. Integrative analysis supports a new species of the *Oecomys catherinae* complex (Rodentia, Cricetidae) from Amazonia. *Journal of Mammalogy* **102**, 69–89 (2021).
47. Sánchez-Vendizú, P., Pacheco, V. & Vivas-Ruiz, D. An Introduction to the Systematics of Small-Bodied *Neacomys* (Rodentia: Cricetidae) from Peru with Descriptions of Two New Species. *American Museum Novitates* **3913**, 1–38 (2018).
48. Schenk, J. J., Rowe, K. C. & Steppan, S. J. Ecological Opportunity and Incumbency in the Diversification of Repeated Continental Colonizations by Muroid Rodents. *Systematic Biology* **62**, 837–864 (2013).
49. Semedo, T. B. F., Da Silva, M. N. F., Carmignotto, A. P. & Rossi, R. V. Three new species of spiny mice, genus *Neacomys* Thomas, 1900 (Rodentia: Cricetidae), from Brazilian Amazonia. *Systematics and Biodiversity* **19**, 1113–1134 (2021).
50. Steppan, S. J. & Schenk, J. J. Muroid rodent phylogenetics: 900-species tree reveals increasing diversification rates. *PLoS ONE* **12**, e0183070 (2017).
51. Teta, P. & D'Elía, G. Taxonomical notes on the long-clawed mole mice of the genus Geoxus (Cricetidae), with the description of a new species from an oceanic island of southern Chile. *Hystrix, the Italian Journal of Mammalogy* **27**, (2016).
52. Teta, P. *et al.* Uncovering cryptic diversity does not end: a new species of leaf-eared mouse, genus *Phyllotis* (Rodentia, Cricetidae), from Central Sierras of Argentina. *Mammalia* **86**, 393–405 (2022).
53. Timm, R. M., Pine, R. H. & Hanson, J. D. A new species of *Tanyuromys* Pine, Timm, and Weksler, 2012 (Cricetidae: Oryzomyini), with comments on relationships within the Oryzomyini. *Journal of Mammalogy* **99**, 608–623 (2018).
54. Weksler, M. Phylogeny of Neotropical oryzomyine rodents (Muridae: Sigmodontinae) based on the nuclear IRBP exon. *Molecular Phylogenetics and Evolution* **29**, 331–349 (2003).
55. Zeballos, H., Medina, C. E., Rico-Cernohorska, A. & Salazar-Bravo, J. Una especie nueva de *Oxymycterus* (Cricetidae: Sigmodontinae: Akodontini) del sureste de Perú. *Mastozool. Neotrop* **28**, 001–019 (2021).

**List of references revised to compiled the occurrences data base of sigmodontines used in biogeographic analyses.**

1. Abreu-Júnior, E. F. de & Percequillo, A. R. Small mammals of the Estação Ecológica de Bananal, southeastern Atlantic Forest, Brazil, with description of a new species of *Brucepattersonius* (Rodentia, Sigmodontinae). *Arq. Zool.* **50**, 1–116 (2019).
2. Almendra, A. L., Rogers, D. S. & González-Cózatl, F. X. Molecular phylogenetics of the *Handleyomys chapmani* complex in Mesoamerica. *Journal of Mammalogy* **95**, 26–40 (2014).
3. Bonvicino, C. R., Casado, F. & Weksler, M. A new species of *Cerradomys* (Mammalia: Rodentia: Cricetidae) from Central Brazil, with remarks on the taxonomy of the genus. *Zoologia (Curitiba)* **31**, 525–540 (2014).
4. Brace, S., Turvey, S. T., Weksler, M., Hoogland, M. L. P. & Barnes, I. Unexpected evolutionary diversity in a recently extinct Caribbean mammal radiation. *Proc. R. Soc. B.* **282**, 20142371 (2015).
5. Brito, J. *et al.* Diversidad insospechada en los Andes de Ecuador: Filogenia del grupo “cinereus” de Thomasomys y descripción de una nueva especie (Rodentia, Cricetidae). *Mastozool. Neotrop* **26**, 308–330 (2019).
6. Brito, J. *et al.* A new genus of oryzomyine rodents (Cricetidae, Sigmodontinae) with three new species from montane cloud forests, western Andean cordillera of Colombia and Ecuador. *PeerJ* **8**, e10247 (2020).
7. Brito, J. *et al.* A new species of spiny mouse, genus *Neacomys* (Cricetidae: Sigmodontinae) from cordillera del Cóndor, Ecuador. *Mastozool. Neotrop* **28**, 001–022 (2021).
8. Brito, J. *et al.* Unlocking Andean sigmodontine diversity: five new species of *Chilomys* (Rodentia: Cricetidae) from the montane forests of Ecuador. *PeerJ* **10**, e13211 (2022).
9. Brito, J. M. *et al.* New species of arboreal rat of the genus *Rhipidomys* (Cricetidae, Sigmodontinae) from Sangay National Park, Ecuador. *Neotropical Biodiversity* **3**, 65–79 (2017).
10. Brito, J., Vaca-Puente, S., Koch, C. & Tinoco, N. Discovery of the first Amazonian *Thomasomys* (Rodentia, Cricetidae, Sigmodontinae): a new species from the remote Cordilleras del Cóndor and Kutukú in Ecuador. *Journal of Mammalogy* **102**, 615–635 (2021).
11. Christoff, A. U. *et al.* A new species of *Juliomys* (Rodentia, Cricetidae, Sigmodontinae) from the Atlantic Forest of Southern Brazil. *Journal of Mammalogy* **97**, 1469–1482 (2016).
12. Colmenares-Pinzón, J. E. Calling for a reassessment of rodent diversity in Colombia: description of a new species of *Neacomys* (Cricetidae: Oryzomyini) from the Magdalena Valley, with a new phylogenetic hypothesis for the genus and comments on its diversification. *Zootaxa* **4920**, 451–494 (2021).
13. D'Elía, G., Teta, P., Upham, N. S., Pardiñas, U. F. J. & Patterson, B. D. Description of a new soft-haired mouse, genus *Abrothrix* (Sigmodontinae), from the temperate Valdivian rainforest. *Journal of Mammalogy* **96**, 839–853 (2015).
14. Dias, D. Sistemática molecular, biogeografia e diversificação de *Brucepattersonius* (Rodentia: Sigmodontinae). Masters Dissertation. Universidade Federal do Espírito Santo, Vitória (2016).
15. do Prado, J. R. & Percequillo, A. R. Systematic Studies of the Genus *Aegialomys* Weksler et al., 2006 (Rodentia: Cricetidae: Sigmodontinae): Geographic Variation, Species Delimitation, and Biogeography. *J Mammal Evol* **25**, 71–118 (2018).
16. Fernandes Semedo, T. B. *et al.* Systematics of Neotropical Spiny Mice, Genus *Neacomys* Thomas, 1900 (Rodentia: Cricetidae), from Southeastern Amazonia, with Descriptions of Three New Species. *American Museum Novitates* **2020**, 1 (2020).
17. Gonçalves, P. R. & Oliveira, J. A. D. An integrative appraisal of the diversification in the Atlantic forest genus *Delomys* (Rodentia: Cricetidae: Sigmodontinae) with the description of a new species. *Zootaxa* **3760**, 1 (2014).
18. Gonzales, F. N., Arce Merma, A. & Zeballos, H. Rango de extensión de la rata acuática peruana *Neusticomys peruviensis* (Rodentia: Cricetidae) en Perú. *Rev peru biol* **24**, 413 (2017).
19. Hanson, J. D. *et al.* A new species of fish-eating rat, genus *Neusticomys* (Sigmodontinae), from Ecuador. *Zool. Stud.* **54**, 49 (2015).
20. Hurtado, N. & D'Elía, G. A new species of long-tailed mouse, genus *Oligoryzomys* Bangs, 1900 (Rodentia: Cricetidae), from the Bolivian Yungas. *Zootaxa* **4500**, 341 (2018).
21. Hurtado, N. & Pacheco, V. Revision of *Neacomys spinosus* (Thomas, 1882) (Rodentia: Cricetidae) with emphasis on Peruvian populations and the description of a new species. *Zootaxa* **4242**, 401 (2017).
22. Hurtado, N. A new species of the genus *Oligoryzomys* (Rodentia: Cricetidae) from Peru. *Journal of Mammalogy* **102**, 931–946 (2021).
23. Inche Arroyo, B. D. Estado taxonómico de *Akodon aerosus baliolus* (Cricetidae: sigmodontinae). *Universidad Nacional Mayor de San Marcos* (2019).
24. Jayat, J. P. *et al.* Establishing the availability of the recently erected binomen *Phyllotis pehuenche* (Rodentia, Cricetidae, Sigmodontinae). *Therya notes* **3**, 110–114 (2022).
25. Jayat, J. P., D’Elía, G., Ortiz, P. E. & Teta, P. A new species of the rodent genus *Necromys* Ameghino (Cricetidae: Sigmodontinae: Akodontini) from the Chaco Serrano grasslands of northwestern Argentina. *Journal of Mammalogy* **97**, 1321–1335 (2016).
26. Jimenez Aguado, C. F. & Pacheco Torres, V. R. A new species of grass mouse, genus *Akodon* Meyen, 1833 (Rodentia, Sigmodontinae) from central Peruvian Yungas. *Therya* **7**, 449–464 (2016).
27. Medina, C. E., López, E., Pino, K., Pari, A. & Zeballos, H. Biodiversidad de la zona reservada Sierra del Divisor (Perú): una visión desde los mamíferos pequeños. *Rev peru biol* **22**, 199 (2015).
28. Moreno Cárdenas, P. A., Tinoco, N., Albuja, L. & Patterson, B. D. A new species of *Rhagomys* (Rodentia, Sigmodontinae) from southeastern Ecuador. *Journal of Mammalogy* **102**, 123–138 (2021).
29. Pacheco, V. *et al.* A revision of *Neusticomys peruviensis* (Rodentia: Cricetidae) with the description of a new subspecies. *Journal of Mammalogy* **101**, 858–871 (2020).
30. Pacheco, V., Rengifo, E. M. & Vivas, D. Una nueva especie de ratón orejón del género *Phyllotis* Waterhouse, 1837 (Rodentia: Cricetidae) del norte del Perú. *Therya* **5**, 481–508 (2014).
31. Pardiñas, U. F. J., Geise, L., Ventura, K. & Lessa, G. A new genus for *Habrothrix angustidens* and *Akodon serrensis* (Rodentia, Cricetidae): again paleontology meets neontology in the legacy of Lund. *Mastozool. Neotrop* **23**(1), 93-115 (2016).
32. Pardiñas, U. F. J., Teta, P., D’Elía, G. & Diaz, G. B. Taxonomic status of *Akodon oenos* (Rodentia, Sigmodontinae), an obscure species from West Central Argentina. *Zootaxa* **2749**, 47 (2011).
33. Pardiñas, U. F. J., Teta, P., Salazar-Bravo, J., Myers, P. & Galliari, C. A. A new species of arboreal rat, genus *Oecomys* (Rodentia, Cricetidae) from Chaco. *Journal of Mammalogy* **97**, 1177–1196 (2016).
34. Patton, J. L., Pardiñas, U. F. J. & D'Elia, G. Mammals of South America, Volume 2: Rodents. University of Chicago Press. 1336 pp (2015).
35. Peçanha, W. T. *et al.* A new species of *Oxymycterus* (Rodentia: Cricetidae: Sigmodontinae) from a transitional area of Cerrado – Atlantic Forest in southeastern Brazil. *Journal of Mammalogy* **100**, 578–598 (2019).
36. Percequillo, A. R. *et al.* The genus *Abrawayaomys* Cunha and Cruz, 1979 (Rodentia: Cricetidae: Sigmodontinae): geographic variation and species definition. *Journal of Mammalogy* **98**, 438–455 (2017).
37. Pinotti, J. D. *et al.* Multiple refugia and glacial expansions in the Tucumane–Bolivian Yungas: The phylogeography and potential distribution modeling of *Calomys fecundus* (Thomas, 1926) (Rodentia: Cricetidae). *J. Zool. Syst. Evol. Res.* **58**, 1359–1373 (2020).
38. Prado, J. R., Knowles, L. L. & Percequillo, A. R. A new species of South America marsh rat (*Holochilus* , Cricetidae) from northeastern Brazil. *Journal of Mammalogy* **102**, 1564–1582 (2021).
39. Rengifo, E. M. & Pacheco, V. M. Taxonomic revision of the Andean leaf-eared mouse, *Phyllotis andium* Thomas 1912 (Rodentia: Cricetidae), with the description of a new species. *Zootaxa* **4018**, 349 (2015).
40. Rengifo, E. M. *et al.* Andean non‐volant small mammals: A dataset of community assemblages of non‐volant small mammals from the high Andes. *Ecology* **103**, (2022).
41. Rocha, R. G. *et al.* Cryptic diversity in the Oecomys roberti complex: revalidation of *Oecomys tapajinus* (Rodentia, Cricetidae). *Journal of Mammalogy* **99**, 174–186 (2018).
42. Ruelas, D. & Pacheco, V. A new species of Thomasomys Coues, 1884 (Rodentia: Sigmodontinae) from the montane forests of northern Peru with comments on the "aureus" group. *Rev peru biol* **28**, e19912 (2021).
43. Ruelas, D., Pacheco, V., Inche, B. & Tinoco, N. A preliminary review of *Nephelomys albigularis* (Tomes, 1860) (Rodentia: Cricetidae), with the description of a new species from the Peruvian montane forests. *Zootaxa* **5027**, 175–210 (2021).
44. Saldanha, J. & Rossi, R. V. Integrative analysis supports a new species of the *Oecomys catherinae* complex (Rodentia, Cricetidae) from Amazonia. *Journal of Mammalogy* **102**, 69–89 (2021).
45. Sánchez-Vendizú, P., Pacheco, V. & Vivas-Ruiz, D. An Introduction to the Systematics of Small-Bodied *Neacomys* (Rodentia: Cricetidae) from Peru with Descriptions of Two New Species. *American Museum Novitates* **3913**, 1–38 (2018).
46. Semedo, T. B. F., Da Silva, M. N. F., Carmignotto, A. P. & Rossi, R. V. Three new species of spiny mice, genus *Neacomys* Thomas, 1900 (Rodentia: Cricetidae), from Brazilian Amazonia. *Systematics and Biodiversity* **19**, 1113–1134 (2021).
47. Souza, M. M. de, Kubiak, B. B., Maestri, R., Kretschmer, R. & Galiano, D. New record of *Juliomys ossitenuis* (Rodentia, Sigmodontinae) in Santa Catarina state, southern Brazil. *CheckList* **16**, 805–809 (2020).
48. Steppan, S. J. *et al.* Evidence of a population of leaf-eared mice *Phyllotis vaccarum* above 6,000 m in the Andes and a survey of high-elevation mammals. *Journal of Mammalogy* **103**, 776–785 (2022).
49. Teta, P. & D'Elía, G. Taxonomical notes on the long-clawed mole mice of the genus *Geoxus* (Cricetidae), with the description of a new species from an oceanic island of southern Chile. *Hystrix, the Italian Journal of Mammalogy* **27**, (2016).
50. Teta, P. *et al.* Uncovering cryptic diversity does not end: a new species of leaf-eared mouse, genus *Phyllotis* (Rodentia, Cricetidae), from Central Sierras of Argentina. *Mammalia* **86**, 393–405 (2022).
51. Timm, R. M., Pine, R. H. & Hanson, J. D. A new species of *Tanyuromys* Pine, Timm, and Weksler, 2012 (Cricetidae: Oryzomyini), with comments on relationships within the Oryzomyini. *Journal of Mammalogy* **99**, 608–623 (2018).
52. Turvey, S. T., Weksler, M., Morris, E. L. & Nokkert, M. Taxonomy, phylogeny, and diversity of the extinct Lesser Antillean rice rats (Sigmodontinae: Oryzomyini), with description of a new genus and species: extinct west indian rice rats. *Zoological Journal of the Linnean Society* **160**, 748–772 (2010).
53. Zeballos, H., Medina, C. E., Rico-Cernohorska, A. & Salazar-Bravo, J. Una especie nueva de *Oxymycterus* (Cricetidae: Sigmodontinae: Akodontini) del sureste de Perú. *Mastozool. Neotrop* **28**, 001–019 (2021).

**List of references revised to compiled the fossil taxa data used in phylogenetic analyses.**

1. Barbiere, F., Cruz, L. E., Ortiz, P. E. & Pardiñas, U. F. J. A new genus of Sigmodontinae (Mammalia, Rodentia, Cricetidae) from the Pliocene of central Argentina. *Journal of Vertebrate Paleontology* **36**, e1199557 (2016).
2. Machado, L. F., Leite, Y. L. R., Christoff, A. U. & Giugliano, L. G. Phylogeny and biogeography of tetralophodont rodents of the tribe Oryzomyini (Cricetidae: Sigmodontinae). *Zool Scr* **43**, 119–130 (2014).
3. Martin, R.A., Peláez-Compomanes, P., Ronez, C., Barbière, F., Kelly, T. S., Lindsay E. H., Baskin, J. A., Czaplewski, N. J. & Pardiñas, U. F. J. A new genus of cricetid rodent (Rodentia: Cricetidae) from the Clarendonian (late Miocene) of North America and a consideration of sigmodontine origins. *Paludicola* **12,** 298–329 (2020).
4. Ortiz, P. E., Jayat, J. P. & Steppan, S. J. A New fossil phyllotine (Rodentia, Sigmodontinae) from the late Pliocene in the Andes of northern Argentina. *Journal of Vertebrate Paleontology* **32**, 1429–1441 (2012).
5. Ortiz, P. E., Pardiñas, U. F. J. & Steppan, S. J. A new fossil phyllotine (Rodentia: Muridae) from Northwester Argentina and relationships of the *Reithrodon* group. *Journal of Mammalogy* **81**, 37–51 (2000).
6. Pardiñas, U. F. J. Un nuevo sigmodontino (Mammalia: Rodentia) del Plioceno de Argentina y consideraciones sobre e registro fósil de los Phyllotini. *Rev Chil Hist Nat* **70**, 543–555 (1997).
7. Pardiñas, U. F. J. A New Genus of Oryzomyine Rodent (Cricetidae: Sigmodontinae) from the Pleistocene of Argentina. *Journal of Mammalogy* **89**, 1270–1278 (2008).
8. Reig, O. A. Roedores cricétidos del Plioceno superior de la provincia de Buenos Aires (Argentina): Publicación del Museo Municipal de Ciencias Naturales de Mar del Plata “*Lorenzo Scaglia*” v. 2, p. 164–190 (1978).
9. Reig, O. A. A new fossil genus of South American cricetid rodents allied to Wiedomys, with an assessment of the Sigmodontinae. *Journal of Zoology* **192**, 257–281 (1980).
10. Ronez, C., Martin, R. A., Kelly, T. S., Barbière, F. & Pardiñas, U. F. J. A brief critical review of sigmodontine rodent origins, with emphasis on paleontological data. *Mastozoología Neotropical* **28**, e0495.
11. Steppan, S. J. & Pardiñas, U. F. J. Two new fossil muroids (Sigmodontinae: Phyllotini) from the early Pleistocene of Argentina: phylogeny and paleoecology. *Journal of Vertebrate Paleontology* **18**, 640–649 (1998).
12. Zijlstra, J. S., Madern, P. A. & van den Hoek Ostende, L. W. New genus and two new species of Pleistocene oryzomyines (Cricetidae: Sigmodontinae) from Bonaire, Netherlands Antilles. *Journal of Mammalogy* **91**, 860–873 (2010).
13. Zijlstra, J. S. A new oryzomyine (Rodentia: Sigmodontinae) from the Quaternary of Curaçao (West Indies). *Zootaxa* **3534**, 61 (2012).
14. Zijlstra, J. S., McFarlane, D. A., Van Den Hoek Ostende, L. W. & Lundberg, J. New rodents (Cricetidae) from the Neogene of Curaçao and Bonaire, Dutch Antilles. *Palaeontology* **57**, 895–908 (2014).
